# Cation Exchange as a Route to Introduce Magnetism to Hybrid-Improper Polar Phases

**DOI:** 10.1021/acs.inorgchem.5c01951

**Published:** 2025-06-20

**Authors:** Rachel Conway, Fabio Orlandi, Pascal Manuel, Yujie Zhang, P. Shiv Halasyamani, Michael A. Hayward

**Affiliations:** † Department of Chemistry 6396 University of Oxford, South Parks Road, Oxford OX1 3QR, U.K.; ‡ ISIS Facility, Rutherford Appleton Laboratory, Chilton, Oxon OX11 0QX, U.K.; § Department of Chemistry, 14743 University of Houston, 112 Fleming Building, Houston, Texas 77204-5003, United States

## Abstract

The pseudo Ruddlesden–Popper phase Li_2_CaTa_2_O_7_ is converted to ZnCaTa_2_O_7_, FeCaTa_2_O_7_, or CoCaTa_2_O_7_ by reaction with the corresponding transition-metal dichloride. Diffraction data reveal that ZnCaTa_2_O_7_ adopts a polar crystal structure (*P*2*cm*) with the Zn^2+^cations ordered into stripes within the interlayer coordination sites, and the TaO_6_ units adopt an *a* ^–^ *b* ^–^ *c* ^+^/–(*a* ^–^ *b* ^–^)*c* ^+^ tilting pattern. In contrast, FeCaTa_2_O_7_ and CoCaTa_2_O_7_ adopt polar structures (*P*2_1_ *nm*) with the transition-metal cations ordered in a checkerboard pattern within the interlayer coordination sites, and the TaO_6_ units adopt an *a* ^–^ *b* ^–^ *c* ^+^/ *b* ^–^ *a* ^–^ *c* ^+^ tilting pattern. The different polar structures adopted are rationalized on the basis of the size of the interlayer transition-metal cation. On cooling, FeCaTa_2_O_7_ (*T*_N_ = 40 K) and CoCaTa_2_O_7_ (*T*_N_ = 25 K) adopt antiferromagnetically ordered states with spins aligned parallel to the crystallographic stacking axis and arranged in a G-type manner. Close inspection of the NPD data collected from FeCaTa_2_O_7_ at low temperature reveals a diffuse component to the magnetic scattering, which, in combination with magnetization data, suggest a glassy component to the low-temperature magnetic state. Neither FeCaTa_2_O_7_ nor CoCaTa_2_O_7_ shows significant lattice parameter anomalies around their respective Néel temperatures, in contrast to the previously reported manganese analogue MnCaTa_2_O_7_.

## Graphical abstract

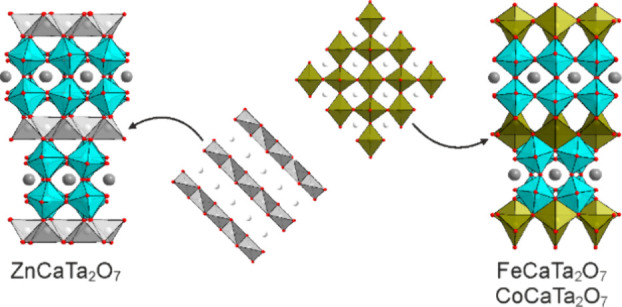

## Introduction

Materials which simultaneously exhibit a spontaneous, switchable electrical polarization (ferroelectricity) and a spontaneous, switchable magnetic polarization (ferromagnetism) are highly desired because such magnetoelectric materials^1–3^ could allow the preparation of novel devices with applications in data manipulation and storage.^4,5^ However, the preparation of novel magnetoelectric materials is challenging because the two constituent behaviors, ferromagnetism and ferroelectricity, are contraindicated.^6^ This is principally because the noncentrosymmetric structural distortions which are a prerequisite of ferroelectric behavior are typically driven by a second-order Jahn–Teller (SOJT) instability^7^ arising from the presence of either octahedrally coordinated d^0^ transition-metal cations (e.g., Ti^4+^ in BaTiO_3_)^8,9^ or *n*s^2^ post-transition-metal cations (e.g., Pb^2+^ in PbZrO_3_)^10,11^ and these species are diamagnetic, so cannot contribute to the desired ferromagnetic behavior.

Recently an alternative ‘trilinear-coupled hybrid-improper’ mechanism for stabilizing ferroelectric structural distortions has been attracting attention.^12,13^ In this scheme, two nonpolar structural distortions (typically the tilting distortions of layered perovskite phases) couple together to stabilize a third, polar distortion (trilinear coupling) which is energetically unfavorable in the absence of the nonpolar distortions. The polar distortion stabilized by this mechanism is not the primary order parameter of the associated ferroelectric phase transition, so polar materials of this type are often referred to as ‘hybrid improper’ ferroelectrics.^14,15^

In principle the hybrid-improper stabilization mechanism does not impose any restrictions on the chemical composition of polar phases, as it is geometric in nature. Thus, paramagnetic species should be easy to incorporate into hybrid-improper ferroelectric phases in an attempt to prepare magnetoelectric materials, as demonstrated by Ca_3_Mn_3_O_7_, the first material theoretically identified as a hybrid-improper ferroelectric.^12^ In practice, however, the requirement to have two distinct octahedral tilting distortions does impose quite strict chemical constraints on the makeup of hybrid improper ferroelectric phases because such highly distorted frameworks are only stable in materials with Goldschmitt tolerance factors (*t* = < A-O>/√2 < B–O>)^16^ smaller than around t = 0.87.^15,17^ This small tolerance factor excludes a large number of combinations of *A*- and *B*-cations, including the majority of Ruddlesden–Popper and Dion-Jacobson phases which can be prepared with paramagnetic transition-metal cations on their *B*-sites.^15^

Recently we have been using the facile exchange reactions of the monovalent cations in *A*’*AB*_2_O_7_ (*A*’ = Cs, Rb) Dion-Jacobson^18–21^ and Li_2_ *AB*_2_O_7_ pseudo Ruddlesden–Popper phases^22^ to prepare metastable layered perovskite oxides with tolerance factors which are small enough to exhibit polar structures stabilized by the hybrid-improper mechanism. Building on this we have also shown that substitution of 2 Li^+^ cations with a divalent paramagnetic ion, such as Mn^2+^, allows magnetic behavior to be introduced.^23,24^ Thus, for example, the hybrid-improper polar phase Li_2_SrTa_2_O_7_ can be converted to MnSrTa_2_O_7_,^23^ a paramagnetic polar material which exhibits signatures of magnetoelectric coupling.

Here we describe the preparation and characterization of FeCaTa_2_O_7_, CoCaTa_2_O_7_ and ZnCaTa_2_O_7_ prepared by analogous cation exchange reactions from Li_2_CaTa_2_O_7_, like the previously reported phase MnCaTa_2_O_7_.^24^

## Experimental Section

### Synthesis of Li_2_CaTa_2_O_7_

Polycrystalline samples of Li_2_CaTa_2_O_7_ were prepared by combining suitable stoichiometric ratios of CaCO_3_ (99.997%) and Ta_2_O_5_ (99%, dried at 900 °C) with a 3% stoichiometric excess of Li_2_CO_3_ (99.99%) to compensate for lithium loss due to volatility at high temperature. The mixture was ground in an agate pestle and mortar, placed in an alumina crucible and heated at 800 °C in air for 12 h. The mixture was then reground, pressed into 13 mm pellets, placed in an alumina crucible and heated to 1200 °C at a rate of 5 °C/min and then held there for 2 h. The sample was then removed from the furnace, reground and pressed into pellets and then directly inserted into a furnace at 1200 °C and heated for 2 h, before being quenched to room temperature. Synchrotron X-ray diffraction data collected from Li_2_CaTa_2_O_7_ could be fit well by the reported structure^25,26^ to yield lattice parameters in good agreement with literature values, as described previously.^24^

### Synthesis of FeCaTa_2_O_7,_ CoCaTa_2_O_7_ and ZnCaTa_2_O_7_

Polycrystalline samples of FeCaTa_2_O_7,_ CoCaTa_2_O_7_ and ZnCaTa_2_O_7_ were prepared via cation exchange reactions from Li_2_CaTa_2_O_7_. Approximately 4 g of Li_2_CaTa_2_O_7_ was combined with 10-mol equiv of anhydrous FeCl_2_ (99.5%), CoCl_2_ (99.7%) or ZnCl_2_ (99.99%). The mixtures were ground in an agate pestle and mortar in an argon-filled glovebox, loaded into Pyrex tubes and sealed under vacuum. The separate samples of FeCaTa_2_O_7,_ CoCaTa_2_O_7_ and ZnCaTa_2_O_7_ were then heated at a ramp rate of 2 °C/min to 350, 360, and 400 °C, respectively, for two periods of 48 h, followed by cooling at 5 °C/min to room temperature. The reaction tube containing ZnCl_2_ was placed vertically in a furnace because ZnCl_2_ has a melting point of 290 °C so the reaction medium is molten at the synthesis temperature. Between heating cycles, the samples were washed with distilled water to remove any unreacted transition-metal chloride, and LiCl byproduct, and then combined with fresh anhydrous transition-metal chloride for the following cycle.

### Characterization

X-ray powder diffraction data were collected using a PANalytical X’pert diffractometer incorporating an X’celerator position-sensitive detector (monochromatic Cu Kα_1_ radiation). High-resolution synchrotron X-ray powder diffraction (SXRD) data were collected using the I11 instrument at the Diamond Light Source Ltd. Diffraction patterns were collected using Si-calibrated X-rays with an approximate wavelength of 0.825 Å from samples, sealed in 0.3 mm diameter borosilicate glass capillaries. Time-of-flight neutron powder diffraction (NPD) data were collected using the WISH diffractometer^27^ located at the ISIS neutron source, from the samples loaded in vanadium cans. Rietveld refinements were performed using the TOPAS Academic (V6).^28^ Second harmonic generation (SHG) response of samples was measured from powder samples with the SHG intensity compared to a standard samples of potassium dihydrogen phosphate (KDP) or AgGaS_2_. No index matching fluid was used in any of the experiments. A detailed description of the experimental setup and process has been reported previously.^29^ DC magnetization data were collected using a Quantum Design MPMS SQUID magnetometer from samples contained in gelatin capsules.

## Results

### Structural Characterization of ZnCaTa_2_O_7_

Powder SHG data collected from ZnCaTa_2_O_7_, using a laser of wavelength 1064 nm, exhibit an activity that is 0.07 times that of a KDP standard, indicating that ZnCaTa_2_O_7_ adopts a noncentrosymmetric structure (Figure S1). SXRD data collected from ZnCaTa_2_O_7_ at room temperature can be indexed using a primitive orthorhombic unit cell (*a* = 5.382 Å, *b* = 5.538 Å, *c* = 19.764 Å) consistent with a √2 × √2 × 1 geometric expansion of an undistorted *n* = 2 Ruddlesden–Popper unit cell, and the data showed no indications of any secondary phases.

NPD data collected from ZnCaTa_2_O_7_ at 100 K could also be indexed using a primitive orthorhombic unit cell (*a* = 5.38116(7) Å, *b* = 5.54170(7) Å, *c* = 19.78286(25) Å). A detailed symmetry analysis of the *n* = 2 Ruddlesden–Popper structure was used to generate a series of distorted, chemically plausible structural models for ZnCaTa_2_O_7_ based on the cooperative tilting of the TaO_6_ units, as described previously.^18^ By considering the reflection conditions observed in the NPD data, and the fact that ZnCaTa_2_O_7_ adopts a noncentrosymmetric crystal structure, it was possible to narrow down the list of possible distorted structures to two candidates: an *a* ^–^ *b* ^–^ *c* ^+^/*b* ^–^ *a* ^–^ *c* ^+^ distorted structure described in space group *P*2_1_ *nm* (#31) or an *a* ^–^ *b* ^–^ *c* ^+^/-(*a* ^–^ *b* ^–^)*c* ^+^ distorted structure described in space group *P*2*cm* (#28). Thus, structural models were constructed for the two distortions based on the structure of Li_2_CaTa_2_O_7_ but with each lithium replaced by 0.5 Zn^2+^ ions. Refinement of these Zn-disordered models against the NPD data revealed the *P*2*cm* symmetry model gave a better fit to the data (*P*2*cm*: wRp = 7.47; *P*2_1_ *nm*: wRp = 13.07).

The possibility of Zn occupational-order was then considered because the two structural models have multiple crystallographically distinct Zn sites: 4 distinct sites in the *P*2*cm* model (2 × 2*a,* 2 × 2*b*) and 2 distinct sites in the *P*2_1_ *nm* model (2 × 4*b*). Ordering the Zn cations onto one of the 4*b* sites within the *P*2_1_ *nm* model would lead to a chequerboard ordering of the Zn cations within the ZnO layers. However, refinement of the Zn site occupancies within the *P*2_1_ *nm* model led to no variation of the disordered distribution of the Zn cations and no improvement to the fit to the data (wRp = 13.07). In contrast, refinement of the Zn site occupancies within the *P*2*cm* model (within the constraint that the ZnCaTa_2_O_7_ composition was maintained) rapidly lead to an occupancy of 0.98(3) for one 2*a* and one 2*b* site with the remaining sites having occupancies of 0.02(3) yielding a structure in which the Zn cations are arranged in stripes within the ZnO layers. This Zn-ordered model fit the NPD data better than the Zn-disorder model (wRp = 4.76) and setting the occupancies of the filled sites to unity made no difference to the fitting statistics. We therefore conclude that the structure of ZnCaTa_2_O_7_ is best described by a Zn-stripe ordered model with an *a* ^–^ *b* ^–^ *c* ^+^/-(*a* ^–^ *b* ^–^)*c* ^+^ tilting distortion described in space group *P*2*cm* as detailed in Table S1, with selected bond lengths given in Table S2, fits to the data shown in Figure S2 and a representation of the structure shown in Figure 1.

**Figure 1:**
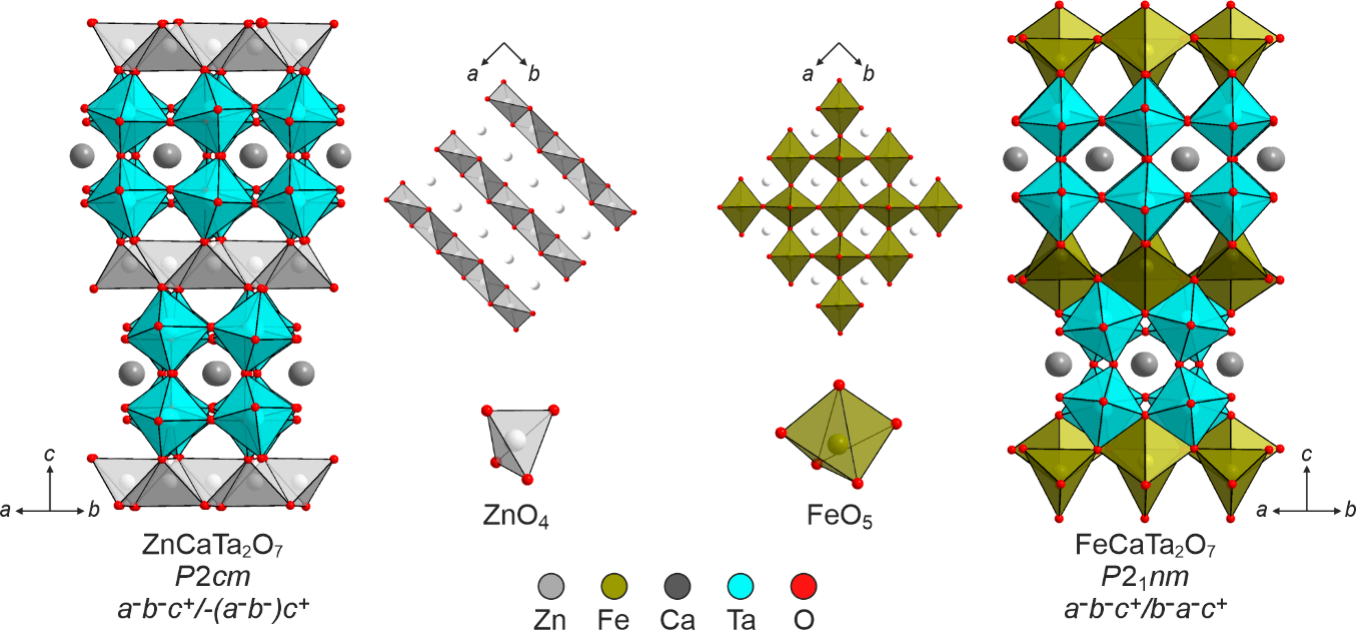
Crystal structures of ZnCaTa_2_O_7_ and FeCaTa_2_O_7_ viewed down their respective [110] axes. Insets show interlayer planes viewed down respective [001] axes.

### Structural Characterization of FeCaTa_2_O_7_

Powder SHG data collected from FeCaTa_2_O_7_, using a laser of wavelength 2090 nm, indicate an activity that is 0.4 times that of an AgGaS_2_ standard, indicating that FeCaTa_2_O_7_ adopts a noncentrosymmetric structure (Figure S1).

SXRD data collected from FeCaTa_2_O_7_ at room temperature could be indexed using a metrically tetragonal unit cell (*a* = 5.513 Å, *c* = 18.658 Å) as could the NPD data collected at 200 K. However, symmetry analysis of the *n* = 2 Ruddlesden–Popper system^18^ revealed that there are no cooperative tilting distortions which yield tetragonal, noncentrosymmetric structures, so collective distortions which yield orthorhombic noncentrosymmetric structures were considered. Of the 4 tilting distortions which yield noncentrosymmetric orthorhombic structures (*a* ^–^ *a* ^–^ *c* ^+^/*a* ^–^ *a* ^–^ *c* ^+^, *A*2_1_ *am*; *a* ^–^ *b* ^–^ *c* ^+^/ *b* ^–^ *a* ^–^ *c* ^+^, *P*2_1_ *nm*; *a* ^–^ *a* ^–^ *c* ^+^/-(*a* ^–^ *a* ^–^ *c* ^+^), *B*2*cm*; *a* ^–^ *b* ^–^ *c* ^+^/-(*a* ^–^ *b* ^–^)*c* ^+^, *P*2*cm*) only those described in space groups *P*2_1_ *nm* and *P*2*cm* have reflection conditions compatible with the NPD data. Thus, structural models were constructed for FeCaTa_2_O_7_ in the space groups *P*2_1_ *nm* and *P*2*cm* based on the structure of Li_2_CaTa_2_O_7_ but with each lithium ion replaced by 0.5 Fe^2+^ cations.

Refinement of these models against the NPD data revealed the *P*2_1_ *nm* symmetry model gave a much better visual and statistical fit (wRp = 7.39) to the data than the *P*2*cm* model (wRp = 12.13), so the *a* ^–^ *b* ^–^ *c* ^+^/ *b* ^–^ *a* ^–^ *c* ^+^ distorted, *P*2_1_ *nm* symmetry model was chosen to describe the structure of FeCaTa_2_O_7_. Close inspection of the *P*2_1_ *nm* model revealed the Fe^2+^ cations can reside on 2 separate 4*b* crystallographic sites, allowing Fe occupationally ordered structures to be described. Refinement of the occupancies of these two sites, under the constraint that the FeCaTa_2_O_7_ composition was conserved, led to a rapid filling of one site and emptying of the other to yield a fully Fe-cation ordered structure. This was accompanied by a significant improvement to the visual and statistical fit (wRp = 4.25). Full details of the refined structure of FeCaTa_2_O_7_ are described in Table S3, with selected bond lengths given in Table S4, fits to the data shown in Figure S3 and a representation of the structure shown in Figure 1.

It should be noted that a cooperative *a* ^–^ *b* ^0^ *c* ^0^/ *b* ^–^ *a* ^0^ *c* ^0^ distortion of an *n* = 2 framework yields a tetragonal structure described in space group *P*4_2_/*mnm*, which can be seen as a special case of the *a* ^–^ *b* ^–^ *c* ^+^/ *b* ^–^ *a* ^–^ *c* ^+^ distortion refined for FeCaTa_2_O_7_. Close inspection of the refined structure of FeCaTa_2_O_7_ reveals that while the *a*-tilt angle (20.3(4)°) is significantly larger than the *b*-tilt (2.1(4)°) or *c*-tilt (5.7(8)°) angles, the latter two tilts have nonzero magnitudes, confirming the choice of a noncentrosymmetric orthorhombic *P*2_1_ *nm* symmetry model, rather than a centrosymmetric tetragonal *P*4_2_/*mnm* model.

### Structural Characterization of CoCaTa_2_O_7_

Powder SHG data collected from CoCaTa_2_O_7_, using a laser of wavelength 2090 nm, indicate an activity that is 0.7 times that of an AgGaS_2_ standard, indicating that CoCaTa_2_O_7_ adopts a noncentrosymmetric structure (Figure S1). SXRD data collected from CoCaTa_2_O_7_ at room temperature could be indexed using a metrically tetragonal unit cell (*a* = 5.516 Å, *c* = 18.59 Å) as could the NPD data collected at 100 K. Using logic analogous to that described above for the structural analysis of FeCaTa_2_O_7_, *a* ^–^ *b* ^–^ *c* ^+^/ *b* ^–^ *a* ^–^ *c* ^+^ and *a* ^–^ *b* ^–^ *c* ^+^/-(*a* ^–^ *b* ^–^)*c* ^+^ distorted structural models were constructed for CoCaTa_2_O_7_ in the space groups *P*2_1_ *nm* and *P*2*cm* respectively, based on the structure of Li_2_CaTa_2_O_7_ but with each lithium ion replaced by 0.5 Co^2+^ cations.

As cobalt has a relatively weak neutron scattering power (2.49 fm)^30^ these structural models were simultaneously refined against both the NPD and SXRD data collected at 100 K. Again, the *P*2_1_ *nm* model gave a better visual and statistical fit (wRp = 4.78) to the data than the *P*2*cm* model (wRp = 6.45), and when the occupancies of the cobalt sites were refined a fully Co occupationally ordered model resulted, again with a significant improvement to the visual and statistical fit (wRp = 2.07). Full details of the refined structure of CoCaTa_2_O_7_ are described in Table S5, with selected bond lengths given in Table S6, fits to the data shown in Figure S4 and S5.

Close inspection of the refined structure of CoCaTa_2_O_7_ revealed the *a*-tilt angle (23.0(3)°) was again significantly larger than the *b*-tilt (1.1(3)°) or *c*-tilt (1.7(7)°) angles. However, all three tilt angles are have nonzero magnitudes, confirming the choice of a noncentrosymmetric orthorhombic *P*2_1_ *nm* symmetry model, rather than a centrosymmetric tetragonal *P*4_2_/*mnm* model for CoCaTa_2_O_7_.

### Magnetic Characterization of FeCaTa_2_O_7_

Magnetization data collected from FeCaTa_2_O_7_ in an applied field of 100 Oe (Figure 2) can be fit by the Curie–Weiss law in the temperature range 150 < *T*/K < 300, yielding values of C = 4.21(3) cm^3^ K mol^–1^ and θ = −107.2(5) K. The observed Curie constant is significantly larger than that expected for a spin-only S = 2 ion (*C*_expected_ = 3 cm^3^ K mol^–1^) suggesting an unquenched orbital contribution to the moment via second-order spin–orbit coupling. The ZFC and FC data diverge weakly below 120 K which we attribute to the presence of a small quantity of Fe_3_O_4_ (Verwey transition *T* ∼ 120 K).^31^ There is a much stronger divergence between ZFC and FC data observed below *T* ∼ 50 K and a local maximum in the ZFC data at *T* = 38 K which is accompanied by an inflection in the FC data. Magnetization data collected as a function of applied field at 300 K are linear and pass through the origin (Figure 2). Analogous data collected at 5 K, after cooling in an applied field of 5 T, are displaced from the origin suggesting a glassy component to the magnetic behavior, although AC susceptibility data collected in the range 35 < *T*/K < 45 show no strong frequency dependence as described in detail in the Supporting Information.

**Figure 2:**
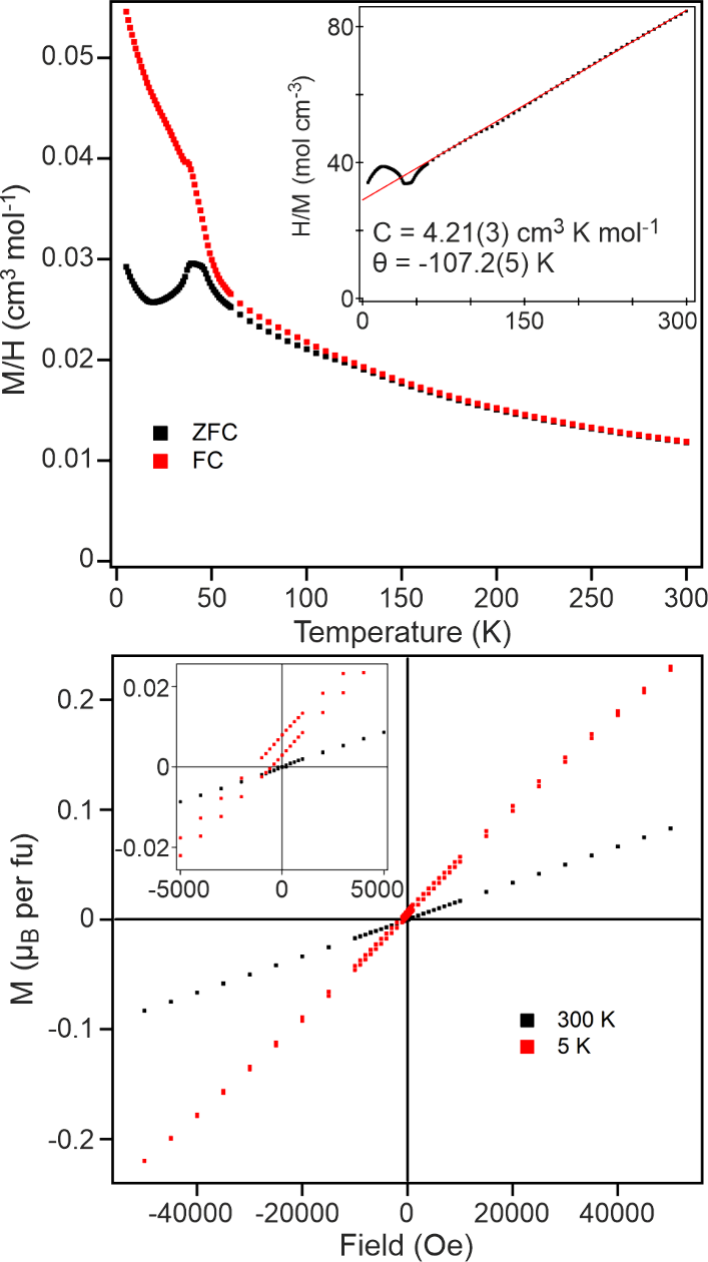
(top) ZFC and FC data collected from FeCaTa_2_O_7_ in an applied field of 100 Oe. Inset shows fit to Curie–Weiss law. (bottom) magnetization-field data collected from FeCaTa_2_O_7_ at 5 and 300 K.

NPD data collected from FeCaTa_2_O_7_ at 1.5 K exhibit a series of additional reflections, not observed in analogous data collected at 200 K (Figure 3a), which are attributed to magnetic order. These additional reflections can be indexed by the crystallographic unit cell, suggesting a propagation vector **k** = (0, 0, 0). A series of magnetic models were constructed on this basis using the ISODISTORT software package^32,33^ and refined against the NPD data. The best fit was achieved using a model obtained by applying the mΓ_2_ magnetic irreducible to the *P*2_1_ *nm* crystallographic structure to yield a model described in magnetic space group *P*2_1_ *n’m’* (#31.127). The components of the ordered moments parallel to the *x*- and *y*-axes refined to zero, within error, while the *z*-component converged to a value of 3.09(1) μ_B_, yielding a model which can be thought of as a G-type antiferromagnetic ordering, as shown in the inset to Figure 3b and described in detail in Table S7.

**Figure 3:**
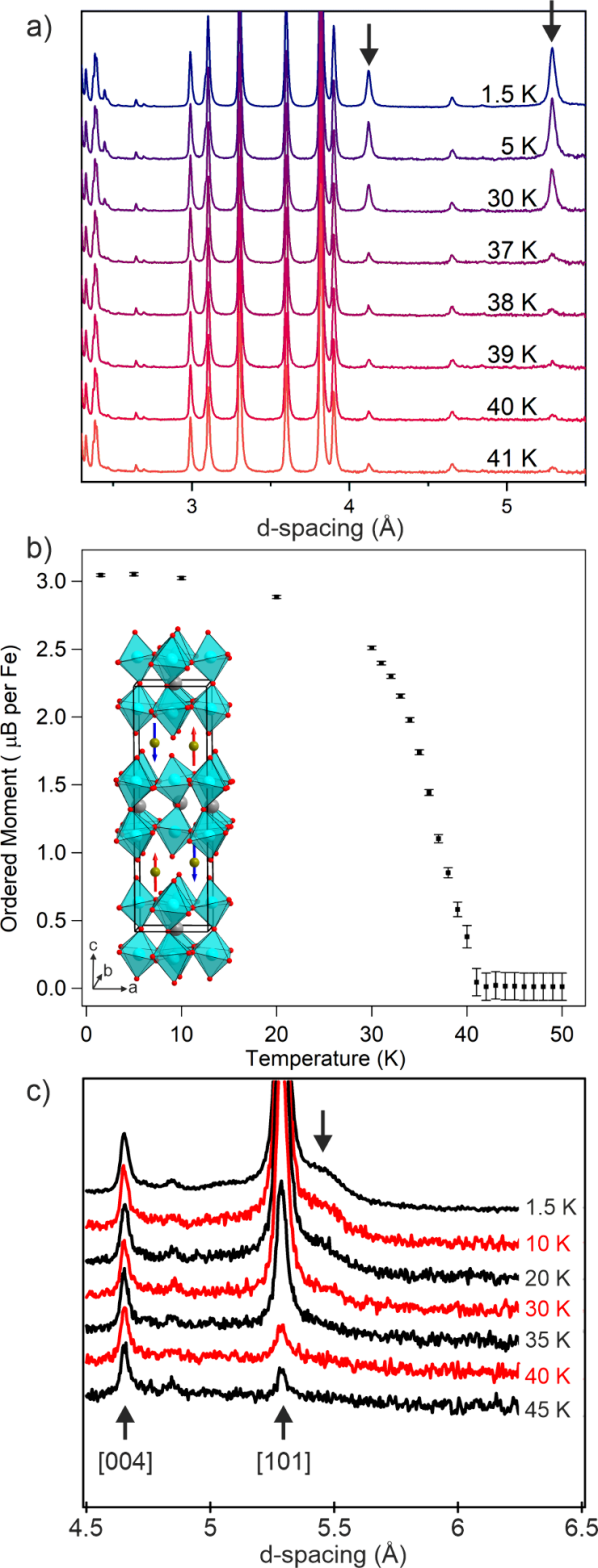
a) NPD data collected from FeCaTa_2_O_7_ at temperature indicated. Arrows indicate magnetic Bragg peaks. b) Plot of ordered magnetic moment as a function of temperature. Inset shows magnetic structure of FeCaTa_2_O_7_ (blue, gray and green and red spheres represent Ta, Ca, Fe, and O respectively).c) Expanded view of NPD data showing diffuse magnetic scattering at d ∼ 5.3 Å.

NPD data collected from FeCaTa_2_O_7_ on warming from 1.5 K can be fit by the same combined nuclear and magnetic model and reveal the ordered moment of the system declines with increasing temperature, as shown in Figure 3a, with no magnetic diffraction intensity observed above 40 K. The temperature dependence of the ordered moment shown in Figure 3b cannot easily be fit by a power law.

Close inspection of the fit of the combined structural and magnetic model to the NPD data collected from FeCaTa_2_O_7_ at 1.5 K reveal a broad, weak diffraction feature centered at d ∼ 5.3 Å, as shown in Figure 3c, which declines in intensity on warming in a manner akin to the main magnetic scattering features. The presence of this diffuse magnetic scattering helps to explain the relatively small size of the observed ordered moment on the Fe centers (3.09 μB compared to an expected value of 4 μB) and when combined with the displaced magnetization-field data shown in Figure 2, suggests there is a disordered or glassy component to the low temperature magnetic state.

### Magnetic Characterization of CoCaTa_2_O_7_

Magnetization data collected from CoCaTa_2_O_7_ in an applied field of 100 Oe (Figure 4) can be fit by the Curie–Weiss law in the temperature range 100 < *T*/K < 300, yielding values of C = 1.988(1) cm^3^ K mol^–1^ and θ = −38.4(8) K, and a temperature independent contribution of 1.75(6) × 10^–3^ cm^3^ mol^–1^. Again, the observed Curie constant is significantly larger than that expected for a spin-only S = ^3^/_2_ ion (*C*_expected_ = 1.875 cm^3^ K mol^–1^) consistent with second-order spin–orbit coupling. On cooling below *T* = 50 K both ZFC and FC data exhibit a sharp increase before diverging at *T* = 28 K. Magnetization-field data collected at 300 K (Figure 4) are linear and pass through the origin, while data collected at 5 K, after cooling in an applied field of 5 T, are sigmoidal and exhibit hysteresis, consistent with canted antiferromagnetic behavior. Analogous data collected on warming show no hysteresis above 28 K and become linear at *T* = 35 K as shown in the Supporting Information.

**Figure 4:**
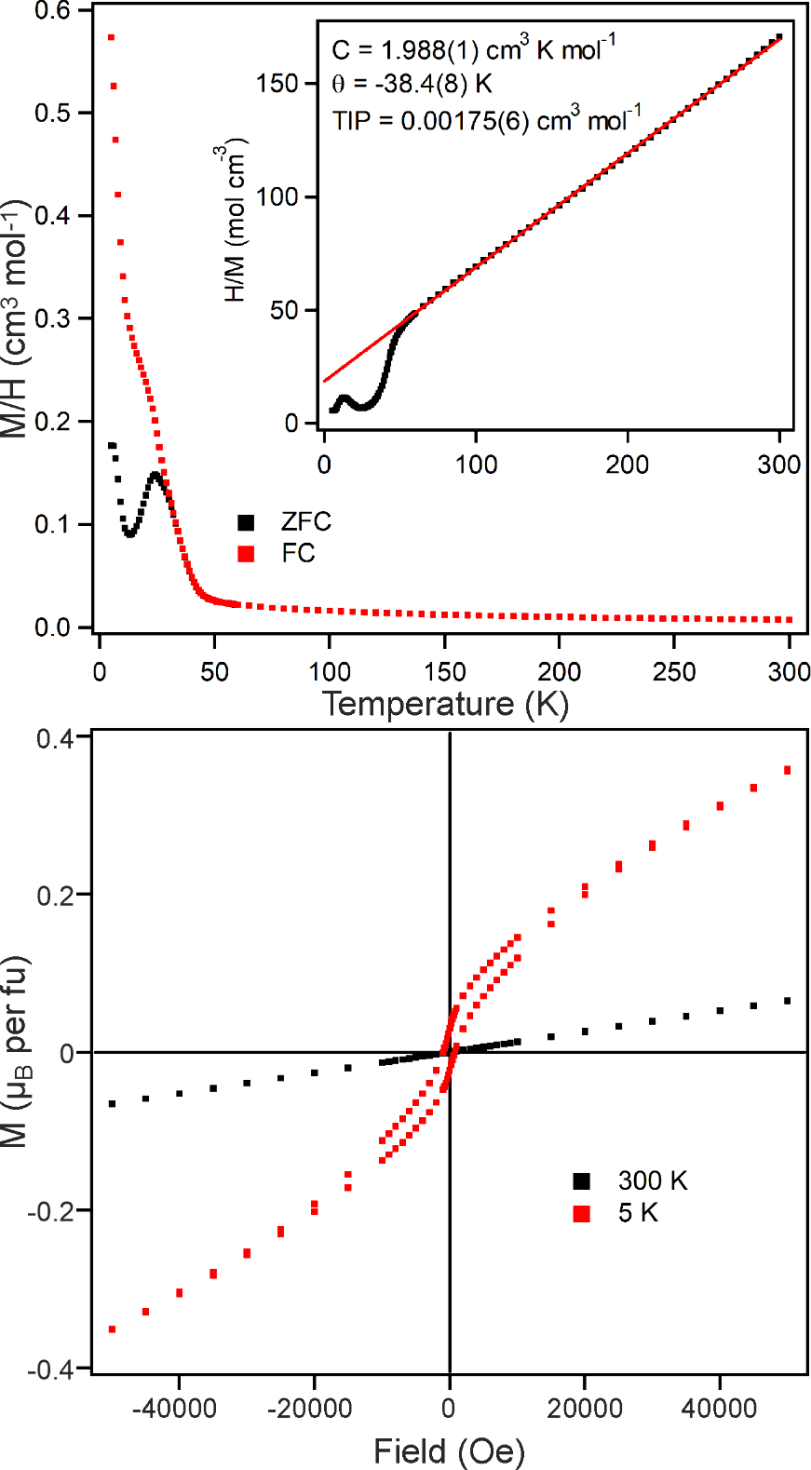
(top) ZFC and FC data collected from CoCaTa_2_O_7_ in an applied field of 100 Oe. Inset shows fit to Curie–Weiss law after subtraction of the temperature independent component. (bottom) magnetization-field data collected from CoCaTa_2_O_7_ at 5 and 300 K.

NPD data collected from CoCaTa_2_O_7_ at 1.5 K exhibit a series of additional reflections not observed in analogous data collected at 100 K (Figure 5a), which are attributed to magnetic order. In common with FeCaTa_2_O_7_ the additional reflections can be indexed using the crystallographic cell and are best fit by a model obtained by applying the mΓ_2_ magnetic irreducible to the *P*2_1_ *nm* crystallographic structure to yield a model described in space group *P*2_1_ *n’m’* (#31.127). On refinement the components of the ordered moments parallel to the *x*- and *y*-axes refined to zero, within error, while the *z*-component converged to a value of 3.07(1) μ_B_, yielding a model directly analogous to that of FeCaTa_2_O_7_, shown in Figure 3 and described in detail in Table S8.

**Figure 5:**
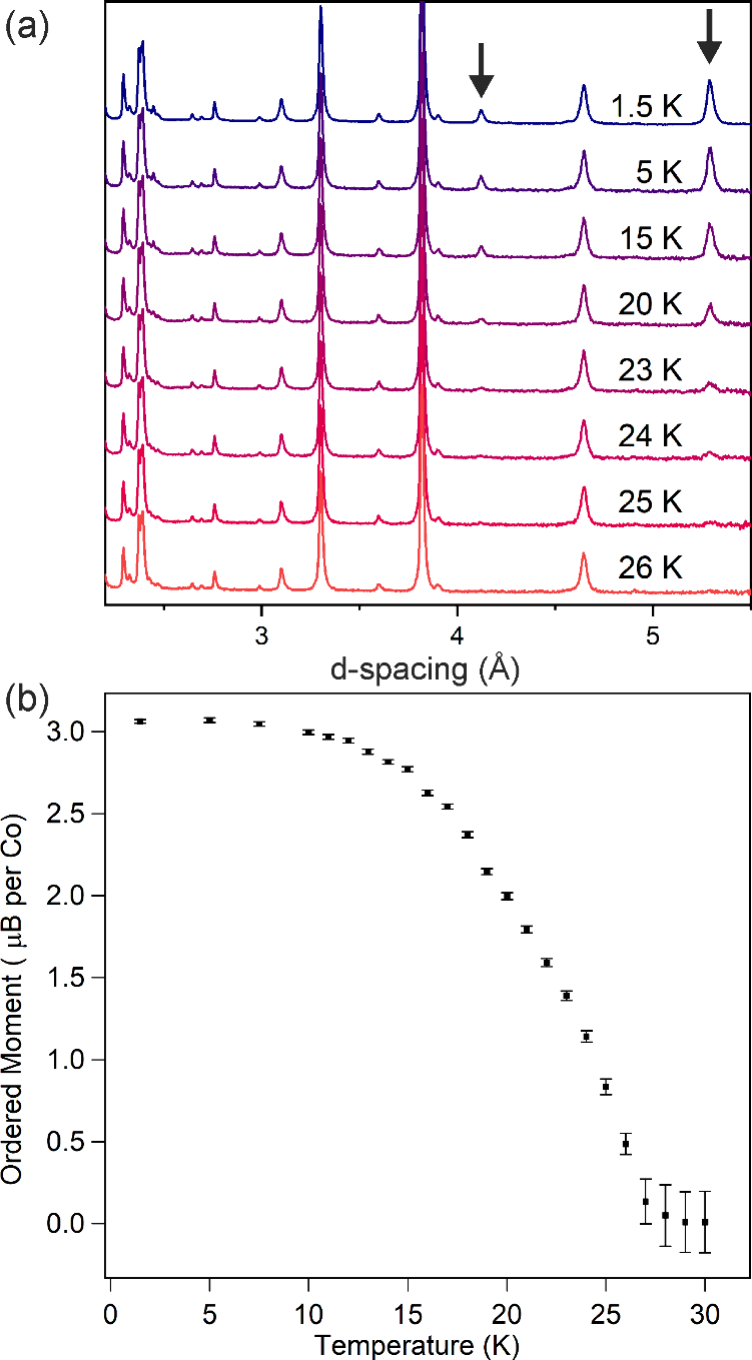
a) NPD data collected from CoCaTa_2_O_7_ at temperature indicated. Arrows indicate magnetic Bragg peaks. b) Plot of ordered magnetic moment as a function of temperature.

NPD data collected from CoCaTa_2_O_7_ on warming from 1.5 K can be fit by the same combined nuclear and magnetic model and reveal the ordered moment of the system declines with increasing temperature, as shown in Figure 5b, with no magnetic diffraction intensity observed above 25 K. Again, the temperature dependence of the ordered moment cannot easily be fit by a power law.

## Discussion

Substitution of the lithium cations in Li_2_CaTa_2_O_7_ with Co^2+^ or Fe^2+^ yields *M*CaTa_2_O_7_ phases which are isostructural with MnCaTa_2_O_7_ ^24^ (space group *P*2_1_ *nm*) in which the transition metal cations adopt a chequerboard vacancy-ordered arrangement within the sites previously occupied by lithium (Figure 1) which stabilizes an *a* ^–^ *b* ^–^ *c* ^+^/ *b* ^–^ *a* ^–^ *c* ^+^ tilting distortion of the TaO_6_ octahedra. In contrast, the Zn substituted phase ZnCaTa_2_O_7_ adopts a structure in which the Zn^2+^ cations are ordered into stripes within the interlayer coordination sites (Figure 1) which stabilizes an *a* ^–^ *b* ^–^ *c* ^+^/-(*a* ^–^ *b* ^–^)*c* ^+^ tilting distortion of the TaO_6_ octahedra, described in space group *P*2*cm.*

Thus, we can see that both the *P*2_1_ *nm* symmetry structure adopted by the Mn, Co and Fe phases, and the *P*2*cm* symmetry structure adopted by ZnCaTa_2_O_7_ contain *a* ^–^ *b* ^–^ *c* ^+^ tilted CaTa_2_O_7_ perovskite layers, with one difference between the two structure types being how the distortions in adjacent layers are oriented relative to each other. In the *P*2_1_ *nm* symmetry, *a* ^–^ *b* ^–^ *c* ^+^/ *b* ^–^ *a* ^–^ *c* ^+^ distorted structure there is a 90° rotation around the *z*-axis of the tilt configuration between adjacent *a* ^–^ *b* ^–^ *c* ^+^ distorted layers. In the *P*2*cm* symmetry *a* ^–^ *b* ^–^ *c* ^+^/-(*a* ^–^ *b* ^–^)*c* ^+^ distorted structure there is an inversion in the direction of the out-of-phase tilts in the *xy*-plane between adjacent *a* ^–^ *b* ^–^ *c* ^+^ distorted layers.

This difference between the adjacent-layer orientation of the CaTa_2_O_7_ tilting distortions can be attributed to the differing interlayer cation ordering schemes of the phases: chequerboard order for *M*CaTa_2_O_7_ (M = Mn, Fe, Co), stripe order for ZnCaTa_2_O_7_. Analogous interlayer cation ordering patterns are observed in LiNdNb_2_O_7_ (stripes) and NaNdNb_2_O_7_ (chequerboard) – two phases prepared via cation exchange from RbNdNb_2_O_7_.^18^ In these *A*NdNb_2_O_7_ phases the differing cation ordering arrangements adopted by the Li and Na phases are rationalized by considering the competition between the desire to minimize *A*-*A* cation repulsion, and the need to optimize the metal–oxygen bonding in the *A*O_4_ local coordination polyhedra. The chequerboard cation ordering in NaNdNb_2_O_7_ consists of sheets of corner-linked NaO_4_ units, and thus minimizes Na–Na cation repulsion in line with Pauling’s third crystallographic rule.^34^ However, this cation arrangement does not allow the size of the NaO_4_ coordination polyhedra to be modified via the tilting of the NbO_6_ octahedra. The stripe-ordered structure of LiNdNb_2_O_7_ consists of sheets of edge-sharing LiO_4_ units and thus has a higher degree of *A*-*A* cation repulsion than the chequerboard arrangement, but crucially in this configuration the tilting of the NbO_6_ units can optimize the size of the LiO_4_ polyhedra. Thus, large cations (i.e., Na^+^) adopt a chequerboard ordered structure to minimize *A*-*A* repulsion, while small cations (i.e., Li^+^) adopt stripe ordered structures because optimizing (shortening) the *A*-O bonds in the local *A*O_4_ units becomes the energetic priority.

This rationalization can be transferred directly to the *M*CaTa_2_O_7_ phases, with the larger *M* ^2+^ cations (Mn, Fe, Co) adopting chequerboard ordered structures and the smaller *M* ^2+^ cations (Zn) adopting stripe ordered structures, and is supported by the observation that the Mn, Fe and Co cations are under bonded in the *M*CaTa_2_O_7_ phases (Mn BVS = +1.85;^24^ Fe BVS = +1.81; Co BVS = +1.80)^35^ while the bonding of the ZnO_4_ units in ZnCaTa_2_O_7_ has been optimized (Zn BVS = +2.01, + 1.99). Thus, it can be seen that the size of the *M* ^2+^ interlayer cations determines the relative orientation of the tilting distortions of adjacent CaTa_2_O_7_ layers.

FeCaTa_2_O_7_, CoCaTa_2_O_7_, and isostructural MnCaTa_2_O_7_ ^24^ adopt analogous magnetically ordered structures at low temperature, with ordering temperatures which scale with the size of the local moment (CoCaTa_2_O_7_: *T*_N_ = 25K, Co^2+^ S = ^3^/_2_ ; FeCaTa_2_O_7_: *T*_N_ = 40K, Fe^2+^ S = 2; MnCaTa_2_O_7_: *T*_N_ = 56K, Mn^2+^ S = ^5^/_2_).^24^ This ‘G-type’ antiferromagnetic order indicates that nearest neighbor antiferromagnetic couplings are the dominant interaction. The signatures of a glassy component to the low-temperature magnetic state of FeCaTa_2_O_7_ can be explained by observing that, in a tetrahedral coordination, Fe^2+^ has a nonspherical e^3^t_2_^3^ electronic configuration (compared to spherical Co^2+^ e^4^t_2_^3^ and Mn^2+^ e^2^t_2_^3^) which could lead to the presence of some ferromagnetic nearest-neighbor couplings which will compete with, and partially frustrate, the dominant antiferromagnetic interactions.

A key reason to prepare cation-exchanged *MAB*_2_O_7_ phases which contain paramagnetic *M*-cations within polar *AB*_2_O_7_ frameworks, is to study the coupling between the magnetic and electrical polarizations in these materials which often becomes apparent at the magnetic ordering temperatures of the phases. For example, MnSrTa_2_O_7_ and MnCaTa_2_O_7_ exhibit large lattice parameter anomalies around 5 K below their respective Néel temperatures (referred to as *T*_A_) which are associated with a step change (MnSrTa_2_O_7_) or local maximum (MnCaTa_2_O_7_) in the Γ_5_^–^ polar distortion mode of the phases and are taken as indications of coupling between the magnetic and electrical polarizations present.^23,24^ Figure 6 shows plots of the lattice parameters of FeCaTa_2_O_7_ and CoCaTa_2_O_7_ as a function of temperature around their respective Néel temperatures. Neither set of data show anomalies analogous to those seen in the MnATa_2_O_7_ phases. Furthermore, if the magnitudes of the X_2_^+^, X_3_^–^ and Γ_5_^–^ distortion modes (required to obtain the *P*2_1_ *nm* symmetry structures of FeCaTa_2_O_7_ and CoCaTa_2_O_7_ from an aristotype *I*4/*mmm* phase) are plotted as a function of temperature over the same ranges (Figures S9 and S10) these also show no significant anomalies, and thus provide no evidence for magnetoelectric coupling in FeCaTa_2_O_7_ or CoCaTa_2_O_7_.

**Figure 6:**
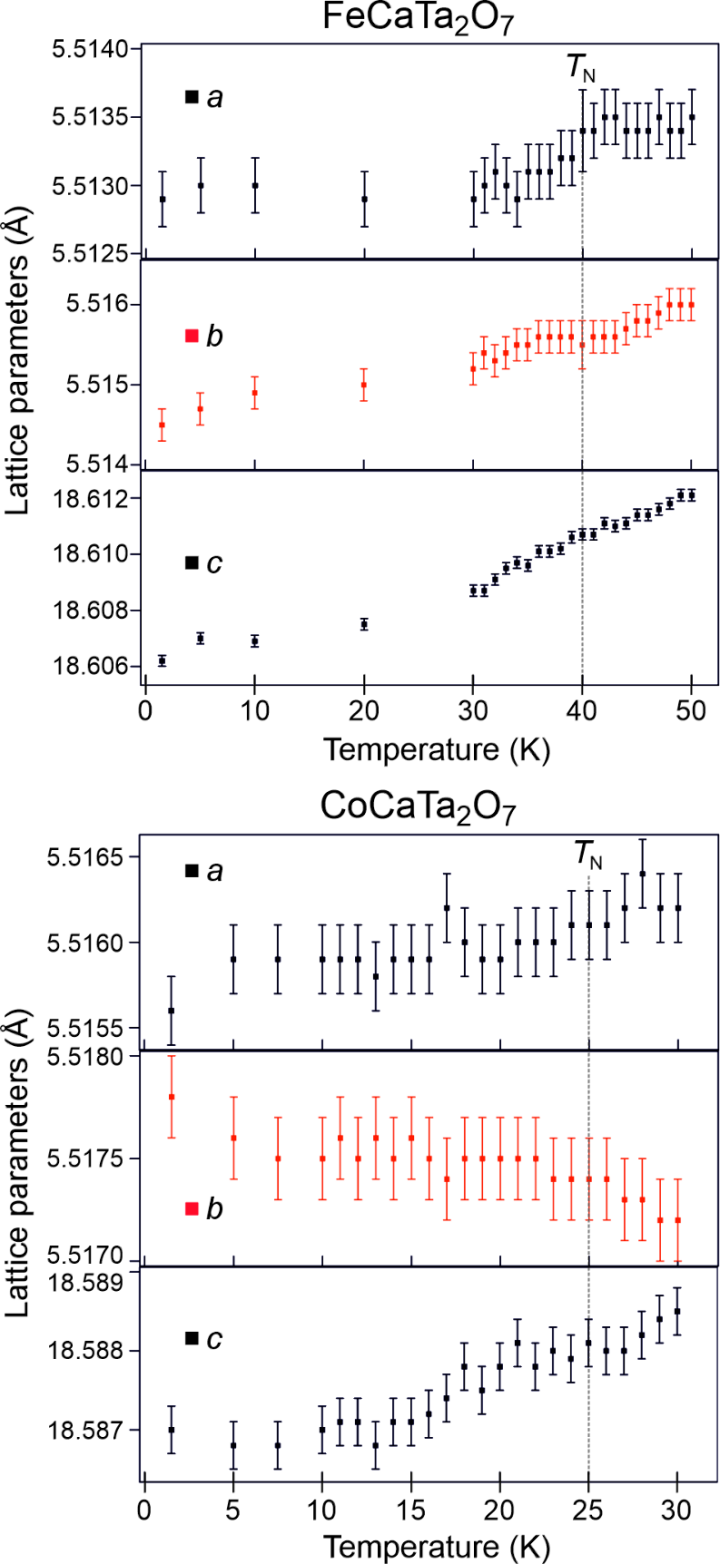
Lattice parameters plotted as a function of temperature for (top) FeCaTa_2_O_7_ and (bottom) CoCaTa_2_O_7_. Dashed lines indicate the Néel temperatures of the two phases.

## Conclusion

FeCaTa_2_O_7_, CoCaTa_2_O_7_ and ZnCaTa_2_O_7_ adopt polar crystal structures consistent with the trilinear-coupled hybrid-improper stabilization mechanism. FeCaTa_2_O_7_ and CoCaTa_2_O_7_ adopt structures described in space group *P*2_1_ *nm*. Comparison of these structures to an *I*4/*mmm*, transition-metal-disordered aristotype phase reveals they are related by the application 4 symmetry lowering distortions with significant magnitude: M_2_^+^ (chequerboard cation order); X_2_^+^(0; *a*) (*a* ^0^ *a* ^0^ *c* ^+^/ *a* ^0^ *a* ^0^ *c* ^+^ tilt); X_3_^–^(*b*; *c*) (*a* ^–^ *b* ^–^ *c* ^0^/ *b* ^–^ *a* ^–^ *c* ^0^ tilt); Γ_5_^–^ (polar distortion), with the presence of the X_2_^+^(0; *a*), X_3_^–^(*b*; *c*) and Γ_5_^–^ modes consistent with the trilinear-coupled hybrid-improper stabilization mechanism. In contrast, ZnCaTa_2_O_7_ adopts a crystal structure described in space group *P*2*cm* which is related to a Zn-disordered, *I*4/*mmm* symmetry aristotype structure by the application only 3 symmetry lowering distortions with significant magnitude: X_4_^–^ (*a*, *b*) (combined Zn stripe-order and *a* ^–^ *b* ^–^ *c* ^0^/-(*a* ^–^ *b* ^–^)*c* ^0^ tilt); X_2_^+^(*a*; 0) (*a* ^0^ *a* ^0^ *c* ^+^/ *a* ^0^ *a* ^0^ *c* ^+^ tilt) and Γ_5_^–^ (polar distortion) which are also symmetry compatible with a trilinear coupling hybrid-improper stabilization mechanism for the observed polar structure.

FeCaTa_2_O_7_ and CoCaTa_2_O_7_ adopt ordered antiferromagnetic states at low temperature with a G-type arrangement, directly analogous to that of MnCaTa_2_O_7_. However, in contrast to the Mn phase, neither FeCaTa_2_O_7_ nor CoCaTa_2_O_7_ show lattice parameter anomalies close to their respective Néel temperatures, and thus show no evidence for magnetoelectric coupling. This lack of magnetoelectric coupling is puzzling given the behavior of the related phases MnCaTa_2_O_7_ and MnSrTa_2_O_7_, and suggests the interactions between the electrical and magnetic polarizations in these phases are subtle and may depend on a further, as yet unidentified parameter.

## Supplementary Material



## Notes

SHG data from all samples. Complete details of refined structures of ZnCaTa_2_O_7_, FeCaTa_2_O_7_ and CoCaTa_2_O_7_ including selected bond lengths. AC susceptibility of FeCaTa_2_O_7_ and magnetization-filed isotherms of CoCaTa_2_O_7_ (PDF)
