# Supplementary material for: Cation Exchange as a Route to Introduce Magnetism to Hybrid-Improper Polar Phases
Source: Inorg Chem. 2025 Jun 20;64(26):13438–45. doi: 10.1021/acs.inorgchem.5c01951 (PMC12239072; doi:10.1021/acs.inorgchem.5c01951)
Supplement: Supplementary file 1 [file ic5c01951_si_001.pdf]

Cation exchange as a route to introduce magnetism to hybrid-improper polar phases.

Rachel Conway[†], Fabio Orlandi[‡], Pascal Manuel[‡], Yujie Zhang[§], P. Shiv Halasyamani[§], and Michael A. Hayward[†]*

[†] Department of Chemistry, University of Oxford, Inorganic Chemistry Laboratory, South Parks Road, Oxford, OX1 3QR, UK.

[‡] ISIS Facility, Rutherford Appleton Laboratory, Chilton, Oxon OX11 0QX, UK.

[§] Department of Chemistry, University of Houston, 112 Fleming Building, Houston, Texas 77204-5003, USA.

*michael.hayward@chem.ox.ac.uk

Supporting Information

Table of Contents

1. Second Harmonic Generation Data.

Figure S1. Powder SHG data collected from a) $ZnCaTa_2O_7$ and a KDP standard using a laser of wavelength 1064 nm and b) $FeCaTa_2O_7$, $CoCaTa_2O_7$ and an $AgGaS_2$ standard using a laser of wavelength 2090 nm.

2. Structural Characterisation of $ZnCaTa_2O_7$

Table S1. Parameters from the structural refinement of $ZnCaTa_2O_7$ against NPD data collected at 100 K.
Table S2. Selected bond lengths and cation bond valence sums from the structure of $ZnCaTa_2O_7$ refined against NPD data collected at 100 K.
Figure S2. Observed calculated and difference plots from the structural refinement of $ZnCaTa_2O_7$ against NPD data collected at 100 K from the 4 different detector banks of the WISH instrument.

3. Structural Characterisation of $FeCaTa_2O_7$

Table S3. Parameters from the structural refinement of $FeCaTa_2O_7$ against NPD data collected at 200 K.
Table S4. Selected bond lengths and cation bond valence sums from the structure of $FeCaTa_2O_7$ refined against NPD data collected at 200 K.
Figure S3. Observed calculated and difference plots from the structural refinement of $FeCaTa_2O_7$ against NPD data collected at 200 K from the 4 different detector banks of the WISH instrument.

4. Structural Characterisation of $CoCaTa_2O_7$

Table S5. Parameters from the structural refinement of $CoCaTa_2O_7$ against NPD and SXRD data collected at 100 K.
Table S6. Selected bond lengths and cation bond valence sums from the structure of $CoCaTa_2O_7$ refined against NPD and SXRD data collected at 100 K.
Figure S4. Observed calculated and difference plots showing the fit to the NPD data collected at 100 K from $CoCaTa_2O_7$ using the 4 different detector banks of the WISH instrument.
Figure S5. Observed calculated and difference plots showing the fit to the SXRD data collected at 100 K from $CoCaTa_2O_7$

5. Magnetic Characterisation of $FeCaTa_2O_7$

Figure S6. AC susceptibility collected from $FeCaTa_2O_7$ as a function of temperature at frequencies between 10 and 500 Hz.
Table S7. Parameters from the structural and magnetic refinement of $FeCaTa_2O_7$ against NPD data collected at 1.5 K.
Figure S7. Observed calculated and difference plots from the structural and magnetic refinement of $FeCaTa_2O_7$ against NPD data collected at 1.5 K from the 4 different detector banks of the WISH instrument.

6. Magnetic Characterisation of $CoCaTa_2O_7$.

Figure S8. Magnetisation-field data collected from $CoCaTa_2O_7$. Lower panel shows expanded view around zero applied field.

Table S8. Parameters from the structural and magnetic refinement of $CoCaTa_2O_7$ against NPD data collected at 1.5 K.

Figure S9. Observed calculated and difference plots from the structural and magnetic refinement of $CoCaTa_2O_7$ against NPD data collected at 1.5 K from the 4 different detector banks of the WISH instrument.

7. Phase transition behaviour

Figure S10. Lattice parameters, X_2^+ (0, *a*), X_3^- (*b*, *c*) and Γ_5^- distortion mode magnitudes of $FeCaTa_2O_7$ plotted as a function of temperature.

Figure S11. Lattice parameters, X_2^+ (0, *a*), X_3^- (*b*, *c*) and Γ_5^- distortion mode magnitudes of $CoCaTa_2O_7$ plotted as a function of temperature.

1. Second Harmonic Generation Data.

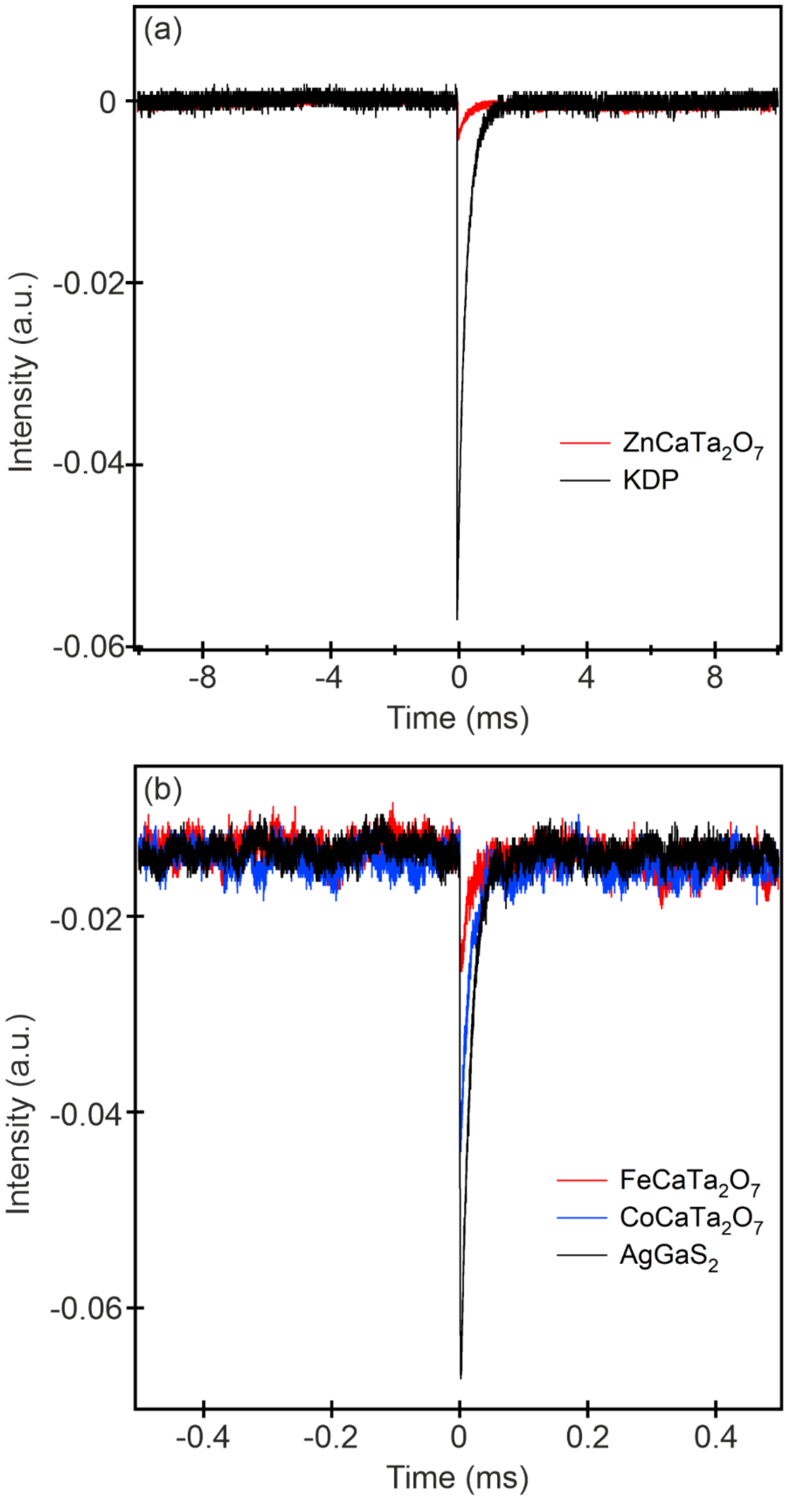

Figure S1. Powder SHG data collected from a) $ZnCaTa_2O_7$ and a KDP standard using a laser of wavelength 1064 nm and b) $FeCaTa_2O_7$, $CoCaTa_2O_7$ and an $AgGaS_2$ standard using a laser of wavelength 2090 nm.

2. Structural Characterisation of $ZnCaTa_2O_7$

Atom	Site	*x*	*y*	*z*	Occ.	B_{iso} / Å^2
Zn1	2*a*	0.9597(20)	0.00000	0	1	0.68(4)
Zn2	2*b*	0.9720(20)	1/2	0	1	0.68(4)
Ca1	2*c*	0.1963(14)	0.2788(15)	¼	1	0.63(7)
Ca2	2*c*	0.7056(15)	0.7930(15)	¼	1	0.63(7)
Ta1	4*d*	0.2231(9)	0.7584(9)	0.1495(3)	1	0.32(2)
Ta2	4*d*	0.7143(9)	0.7480(9)	0.8541(2)	1	0.32(2)
O1	2*c*	0.3003(12)	0.7363(17)	¼	1	0.71(5)
O2	2*c*	0.6583(13)	0.2192(18)	¼	1	0.71(5)
O3	4*d*	0.0100(12)	0.9667(8)	0.8293(3)	1	0.44(4)
O4	4*d*	0.9468(14)	0.5069(9)	0.3268(3)	1	0.44(4)
O5	4*d*	0.5197(12)	0.9424(10)	0.3586(4)	1	0.60(3)
O6	4*d*	0.4279(13)	0.5417(12)	0.8565(4)	1	0.60(3)
O7	4*d*	0.1290(9)	0.7541(14)	0.0552(4)	1	0.71(3)
O8	4*d*	0.8019(10)	0.7468(15)	0.9470(4)	1	0.71(3)
$ZnCaTa_2O_7$ - Space group *P*2*cm* (#28)						
a = 5.38116(7) Å, *b* = 5.54170(7) Å, *c* = 19.78286(25) Å						
Volume = 589.940(13) Å^3						
Formula weight = 579.36 g mol^{-1}, Z = 4						
Radiation source: Neutron Time of Flight, Instrument: WISH						
Temperature: 100 K						
R_p = 4.01 % $_wR_p$ = 4.76 %						

Table S1. Parameters from the structural refinement of $ZnCaTa_2O_7$ against NPD data collected at 100 K.

Cation	Anion	Bond length	BVS
Zn1	O8 × 2	1.945(9)	+ 2.014
	O7 × 2	1.971(9)	
Zn2	O8 × 2	1.950(10)	+ 1.995
	O7 × 2	1.973(9)	
Ca1	O3 × 2	2.306(9)	+ 2.172
	O4 × 2	2.391(9)	
	O2 × 1	2.508(11)	
	O1 × 1	2.597(13)	
	O6 × 2	2.644(9)	
Ca2	O1 × 1	2.204(11)	+ 2.064
	O2 × 1	2.376(13)	
	O5 × 2	2.510(9)	
	O4 × 2	2.552(9)	
	O3 × 2	2.630(9)	
Ta1	O5 × 1	1.901(8)	+ 5.114
	O7 × 1	1.931(9)	
	O3 × 1	1.951(8)	
	O6 × 1	1.999(9)	
	O1 × 1	1.035(5)	
	O4 × 1	2.090(9)	
Ta2	O8 × 1	1.899(9)	+ 5.047
	O6 × 1	1.920(9)	
	O4 × 1	1.963(8)	
	O5 × 1	2.012(8)	
	O3 × 1	2.059(8)	
	O2 × 1	2.089(5)	

Table S2. Selected bond lengths and cation bond valence sums from the structure of $ZnCaTa_2O_7$ refined against NPD data collected at 100 K.

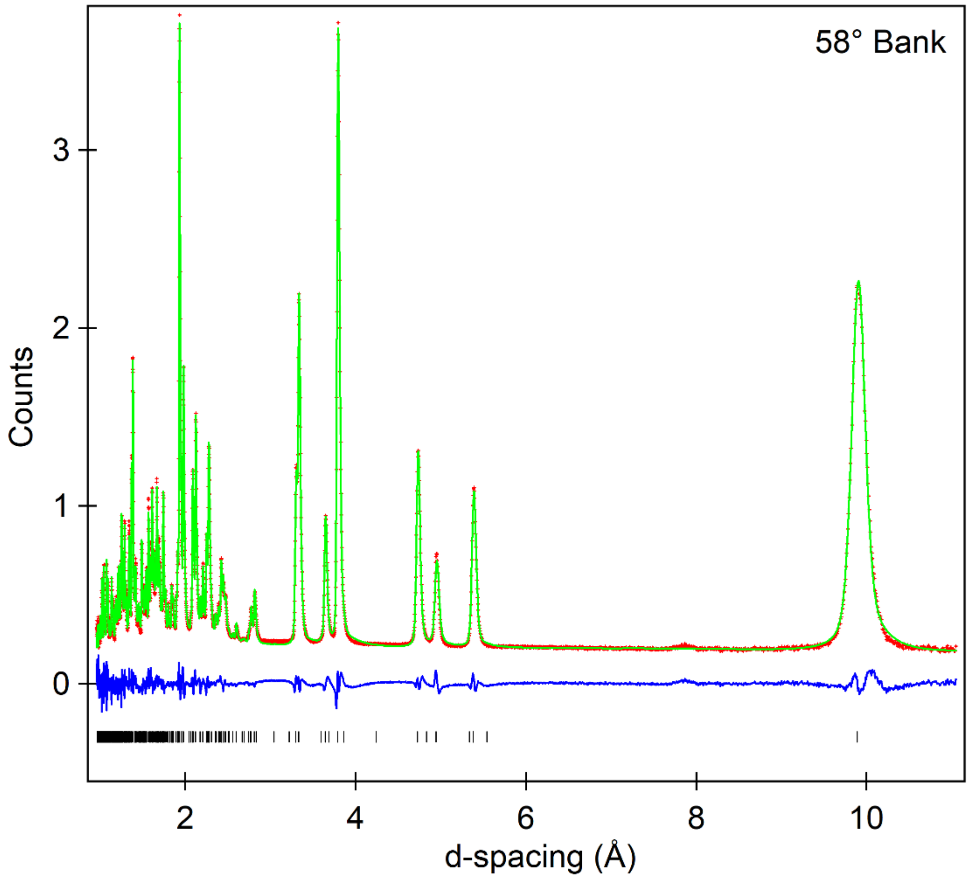
58° Bank
Counts
d-spacing (Å)

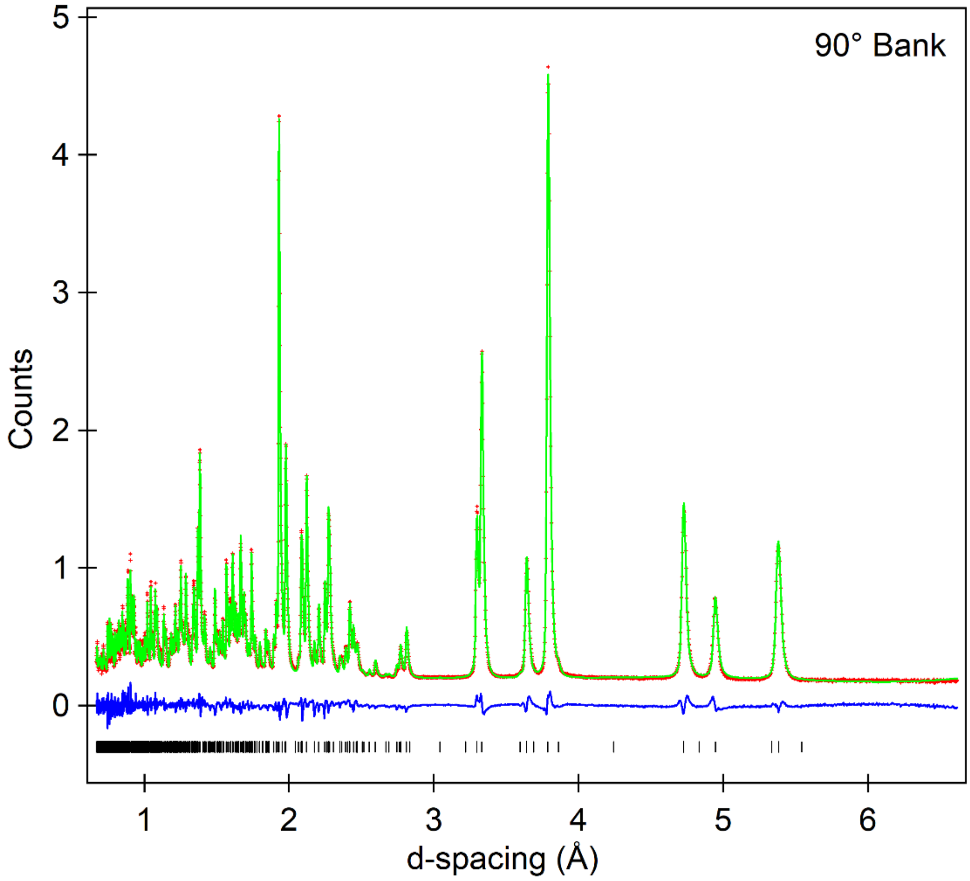
90° Bank
Counts
d-spacing (Å)

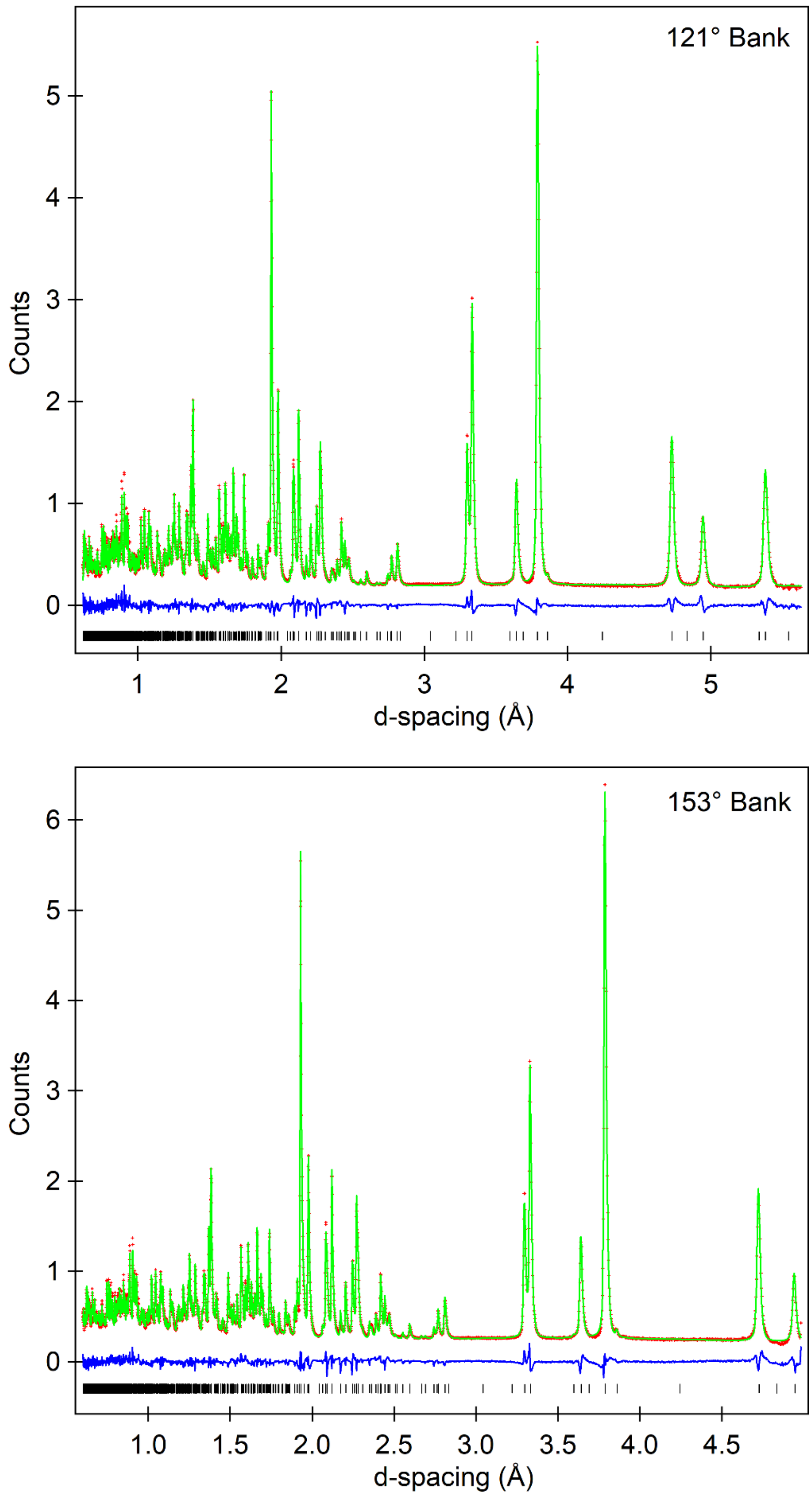

Figure S2. Observed calculated and difference plots from the structural refinement of $ZnCaTa_2O_7$ against NPD data collected at 100 K from the 4 different detector banks of the WISH instrument.

3. Structural Characterisation of $FeCaTa_2O_7$

Atom	Site	*x*	*y*	*z*	Occ.	B_{iso} / Å^2
Fe1	4*b*	0.7615(20)	0.7459(14)	0.26281(8)	1	1.78(3)
Ca1	2*a*	0.0575(16)	0.0152(16)	0	1	0.44(6)
Ca2	2*a*	0.5259(20)	0.5091(17)	0	1	0.44(6)
Ta1	4*b*	0.0188(11)	0.5062(10)	0.89383(38)	1	0.27(2)
Ta2	4*b*	0.5217(10)	0.0027(10)	0.10921(38)	1	0.27(2)
O1	2*a*	0.0547(19)	0.4303(14)	0	1	0.45(4)
O2	2*a*	0.4527(19)	0.0612(16)	0	1	0.45(4)
O3	4*b*	0.2645(24)	0.7385(13)	0.59064(21)	1	0.31(3)
O4	4*b*	0.2544(20)	0.2598(15)	0.42000(9)	1	0.31(3)
O5	4*b*	0.2738(20)	0.2521(15)	0.12419(11)	1	0.51(4)
O6	4*b*	0.2582(27)	0.7666(15)	0.90467(24)	1	0.51(4)
O7	4*b*	0.9838(9)	0.5371(14)	0.79443(49)	1	0.79(3)
O8	4*b*	0.5405(15)	0.0357(16)	0.20819(16)	1	0.79(3)

$FeCaTa_2O_7$ - Space group $P2_1nm$ (#31)

a = 5.5143(1) Å, *b* = 5.5187(1) Å, *c* = 18.64255(18) Å

volume = 567.333(18) Å^3

Formula weight = 569.81 g mol^{-1}, Z = 4

Radiation source: Neutron Time of Flight, Instrument: WISH

Temperature: 200 K

R_p = 4.25 % $_wR_p$ = 4.93 %

Table S3. Parameters from the structural refinement of $FeCaTa_2O_7$ against NPD data collected at 200 K.

Cation	Anion	Bond length	BVS
Fe1	O7 × 1	1.988(12)	+ 1.817
	O8 × 1	2.006(13)	
	O5 × 1	2.103(3)	
	O7 × 1	2.254(12)	
	O8 × 1	2.291(12)	
Ca1	O2 × 1	2.136(15)	+ 2.045
	O1 × 1	2.304(14)	
	O6 × 2	2.473(12)	
	O4 × 2	2.725(13)	
	O3 × 2	2.748(15)	
Ca2	O4 × 2	2.367(11)	+ 1.796
	O2 × 1	2.479(13)	
	O3 × 2	2.537(13)	
	O1 × 1	2.618(15)	
	O6 × 2	2.713(13)	
Ta1	O7 × 1	1.862(15)	+ 5.227
	O3 × 1	1.960(15)	
	O6 × 1	1.970(15)	
	O4 × 1	2.006(13)	
	O5 × 1	2.012(13)	
	O1 × 1	2.045(8)	
Ta2	O8 × 1	1.856(15)	+ 5.199
	O6 × 1	1.960(15)	
	O4 × 1	1.985(12)	
	O5 × 1	1.987(12)	
	O3 × 1	1.998(15)	
	O2 × 1	2.090(8)	

Table S4. Selected bond lengths and cation bond valence sums from the structure of $FeCaTa_2O_7$ refined against NPD data collected at 200 K.

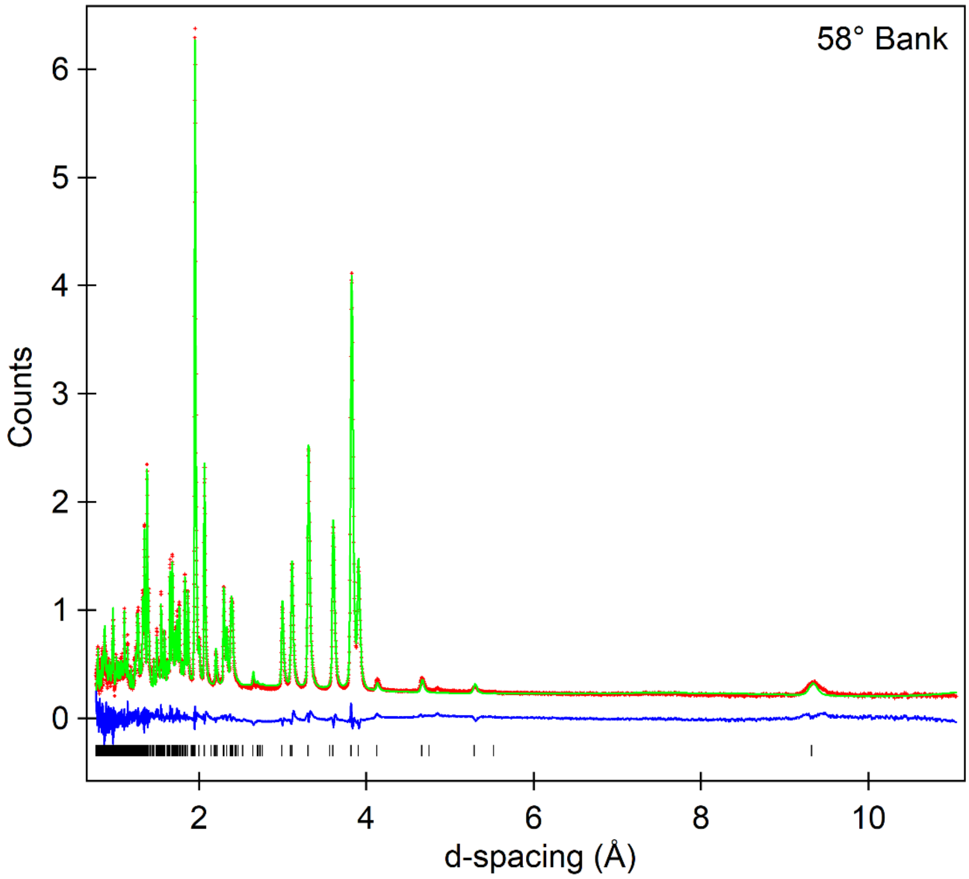

58° Bank
Counts
d-spacing (Å)

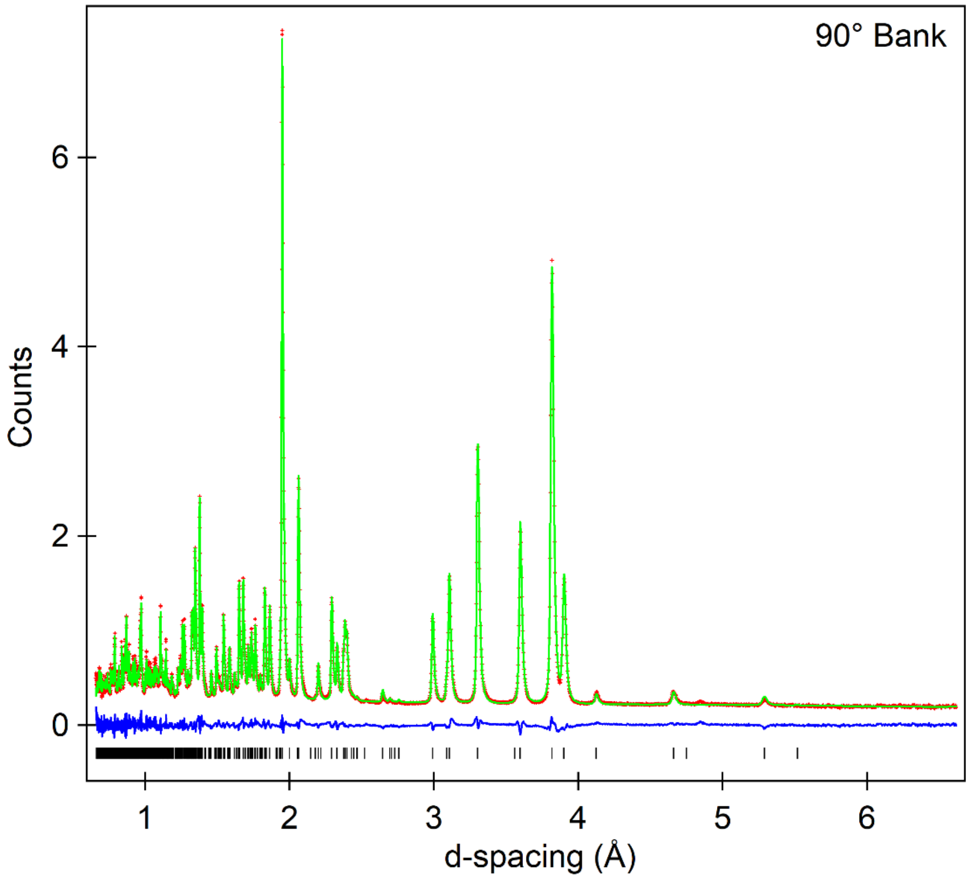

90° Bank
Counts
d-spacing (Å)

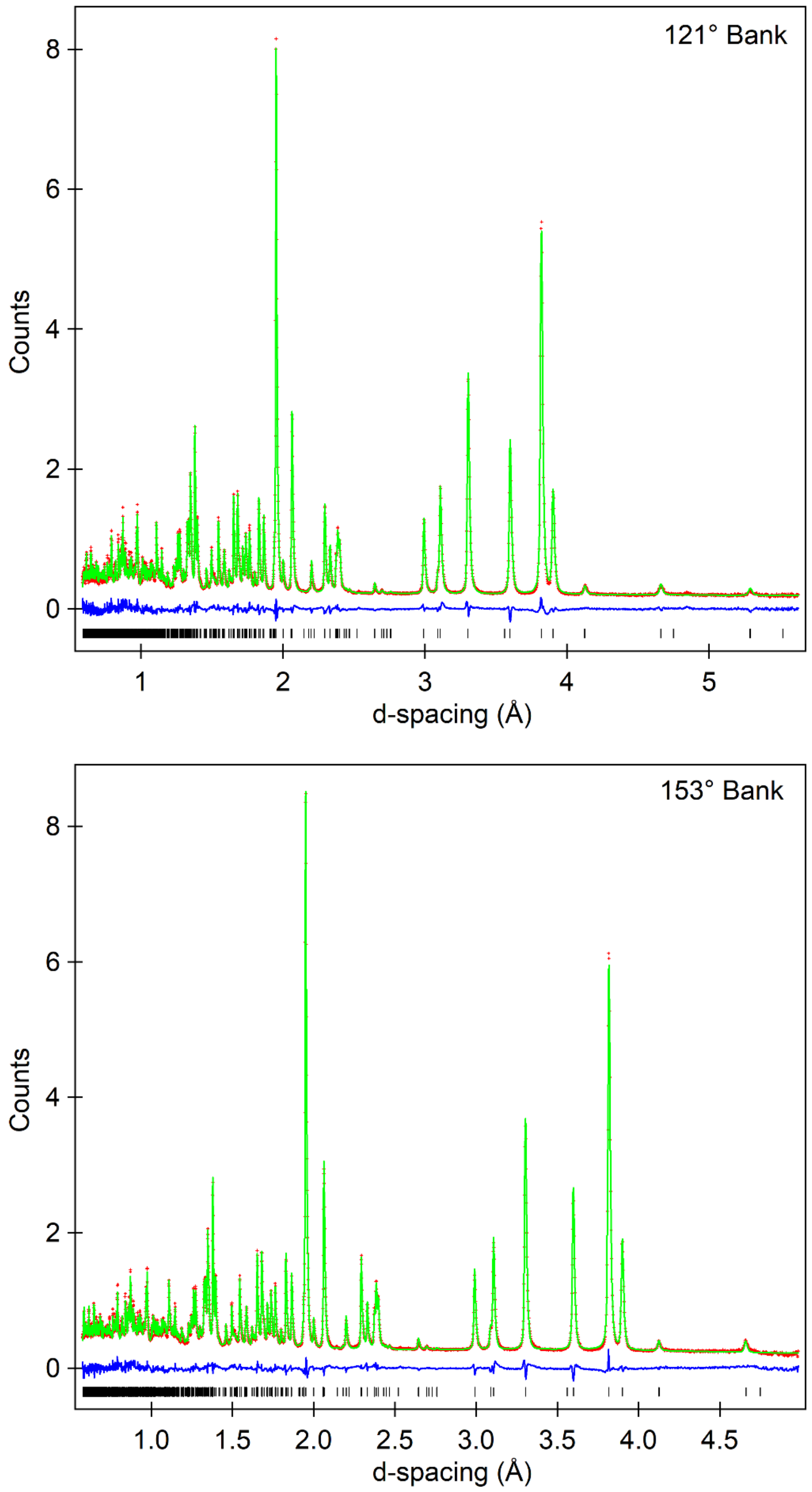

Figure S3. Observed calculated and difference plots from the structural refinement of $FeCaTa_2O_7$ against NPD data collected at 200 K from the 4 different detector banks of the WISH instrument.

4. **Structural Characterisation of $CoCaTa_2O_7$**

Atom	Site	*x*	*y*	*z*	Occ.	B_{iso} / Å^2
Co1	4*b*	0.820(11)	0.7620(123)	0.2331(6)	1	5*
Ca1	2*a*	0.078(11)	0.9854(18)	0	1	0.89(3)
Ca2	2*a*	0.572(11)	0.5108(25)	0	1	0.89(3)
Ta1	4*b*	0.075(11)	0.4964(7)	0.8905(1)	1	0.35(1)
Ta2	4*b*	0.570(11)	0.9999(8)	0.1067(1)	1	0.35(1)
O1	2*a*	0.984(11)	0.5426(18)	0	1	0.56(5)
O2	2*a*	0.611(11)	0.9284(7)	0	1	0.56(5)
O3	4*b*	0.318(11)	0.7460(16)	0.5889(2)	1	0.67(1)
O4	4*b*	0.322(11)	0.2512(18)	0.3720(1)	1	0.67(1)
O5	4*b*	0.320(11)	0.2442(15)	0.0782(1)	1	0.68(1)
O6	4*b*	0.325(10)	0.7441(16)	0.9020(2)	1	0.68(1)
O7	4*b*	0.089(11)	0.4480(10)	0.7939(3)	1	0.98(5)
O8	4*b*	0.521(11)	0.0505(14)	0.2066(4)	1	0.98(5)

$CoCaTa_2O_7$ - Space group $P2_1nm$ (#31)

a = 5.51504(17) Å, b = 5.51841(17) Å, c = 18.59118(22) Å

volume = 565.807(25) Å^3

Formula weight = 572.90 g mol^{-1}, Z = 4

Radiation source: Neutron Time of Flight

Synchrotron X-ray, λ = 0.82381 Å

Temperature: 100 K

R_p = 2.07 % $_wR_p$ = 2.89 %

Table S5. Parameters from the structural refinement of $CoCaTa_2O_7$ against NPD and SXRD data collected at 100 K. Displacement factor of Co fixed at 5 Å^2, as this could not be refined satisfactorily due to weak scattering power of Co.

Cation	Anion	Bond length	BVS
Co1	O8 × 1	1.88(6)	+ 1.803
	O4 × 1	1.954(12)	
	O7 × 1	2.06(6)	
	O7 × 1	2.34(7)	
	O8 × 1	2.35(7)	
Ca1	O5 × 2	2.437(11)	+ 1.635
	O1 × 1	2.497(15)	
	O2 × 1	2.595(12)	
	O6 × 2	2.634(10)	
	O3 × 2	2.643(11)	
Ca2	O1 × 1	2.278(19)	+ 2.052
	O2 × 1	2.313(12)	
	O5 × 2	2.493(14)	
	O3 × 2	2.565(13)	
	O6 × 2	2.614(13)	
Ta1	O7 × 1	1.818(7)	+ 5.224
	O6 × 1	1.949(10)	
	O3 × 1	1.988(10)	
	O4 × 1	2.004(11)	
	O5 × 1	2.023(10)	
	O1 × 1	2.112(4)	
Ta2	O8 × 1	1.898(8)	+ 5.127
	O6 × 1	1.962(10)	
	O3 × 1	1.988(11)	
	O5 × 1	1.999(10)	
	O4 × 1	2.003(11)	
	O2 × 1	2.035(3)	

Table S6. Selected bond lengths and cation bond valence sums from the structure of $CoCaTa_2O_7$ refined against NPD and SXRD data collected at 100 K.

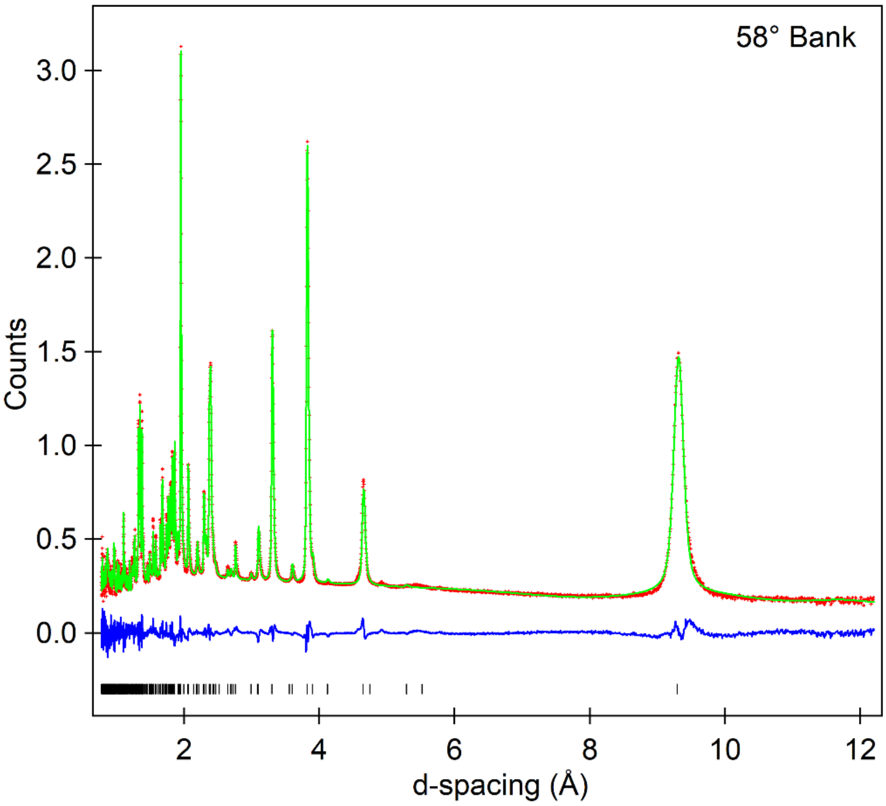
58° Bank
Counts
3.0
2.5
2.0
1.5
1.0
0.5
0.0
2
4
6
8
10
12
d-spacing (Å)

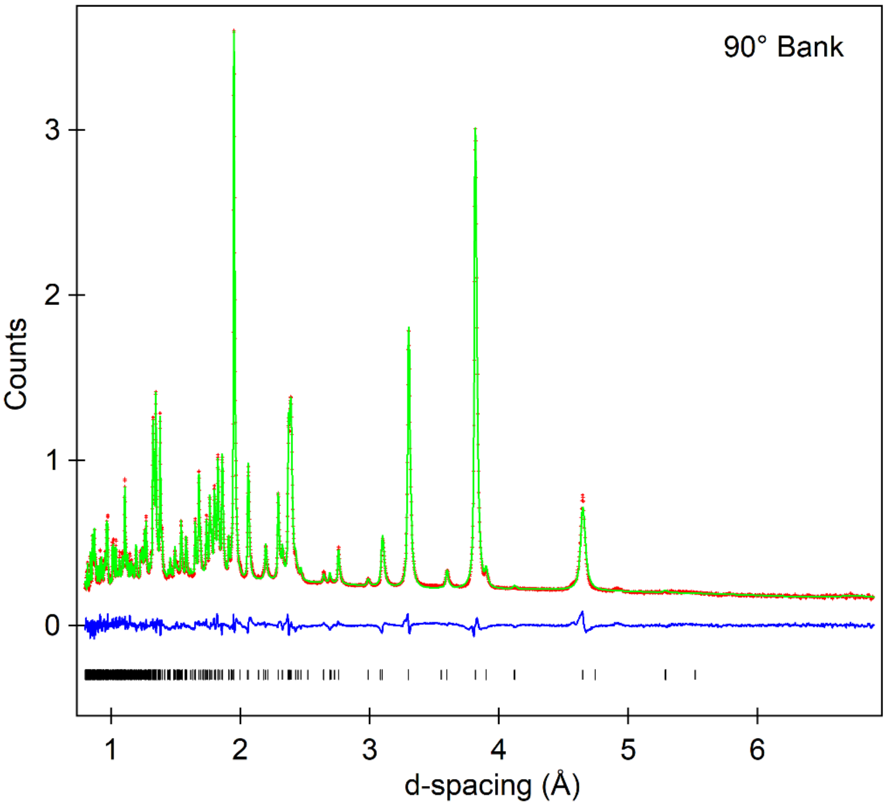
90° Bank
Counts
3
2
1
0
1
2
3
4
5
6
d-spacing (Å)

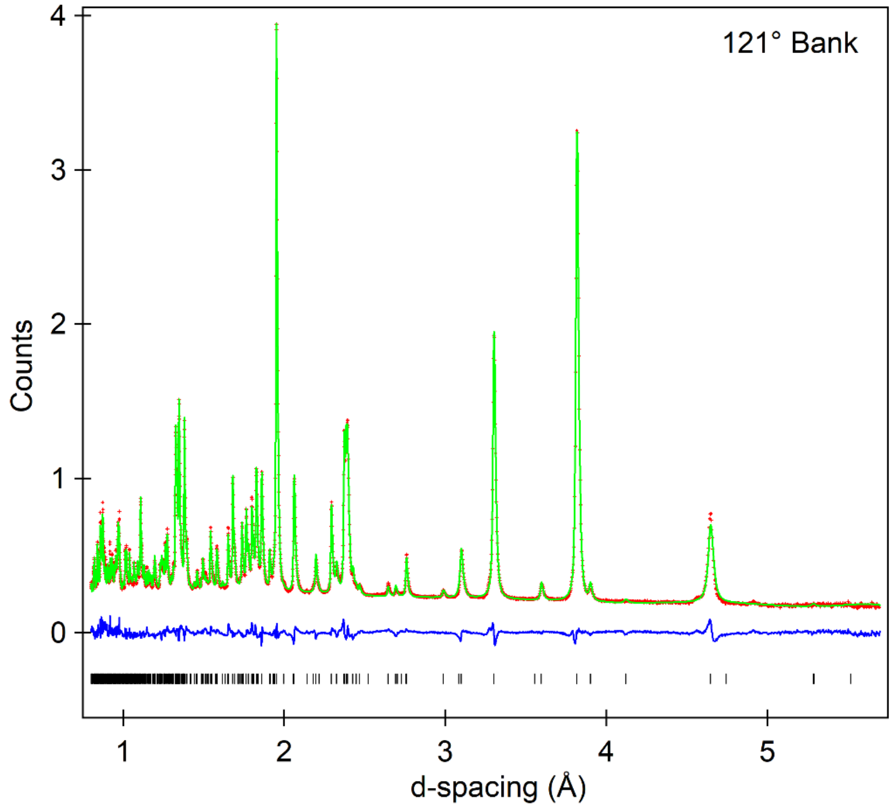

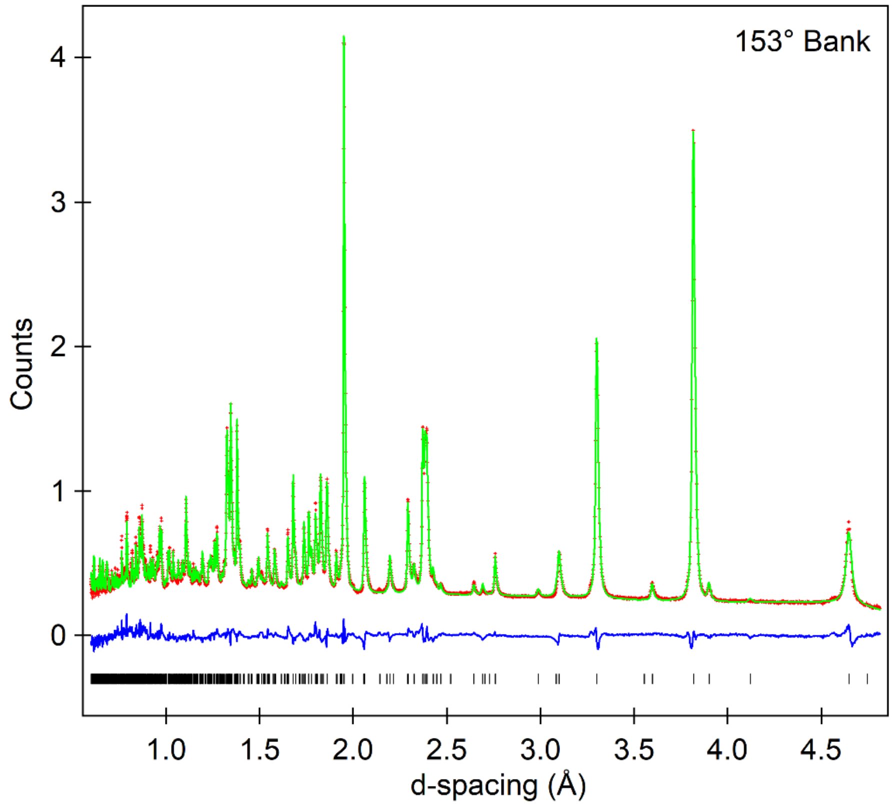

Figure S4. Observed calculated and difference plots showing the fit to the NPD data collected at 100 K from $CoCaTa_2O_7$ using the 4 different detector banks of the WISH instrument.

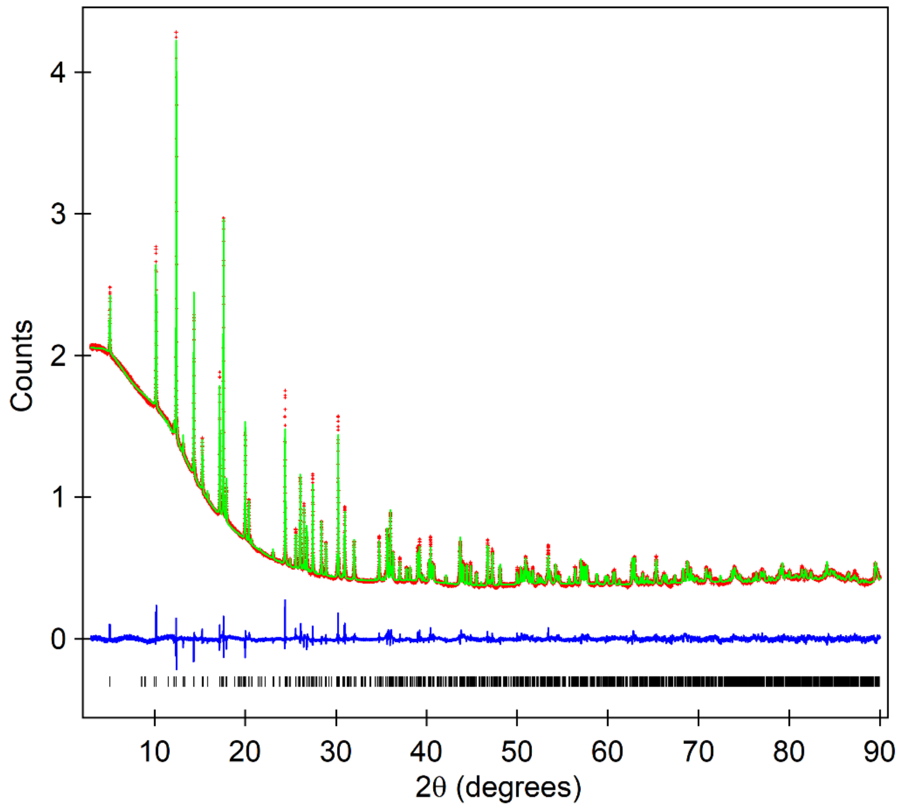

Figure S5. Observed calculated and difference plots showing the fit to the SXRD data collected at 100 K from $CoCaTa_2O_7$

5. **Magnetic Characterisation of $FeCaTa_2O_7$**

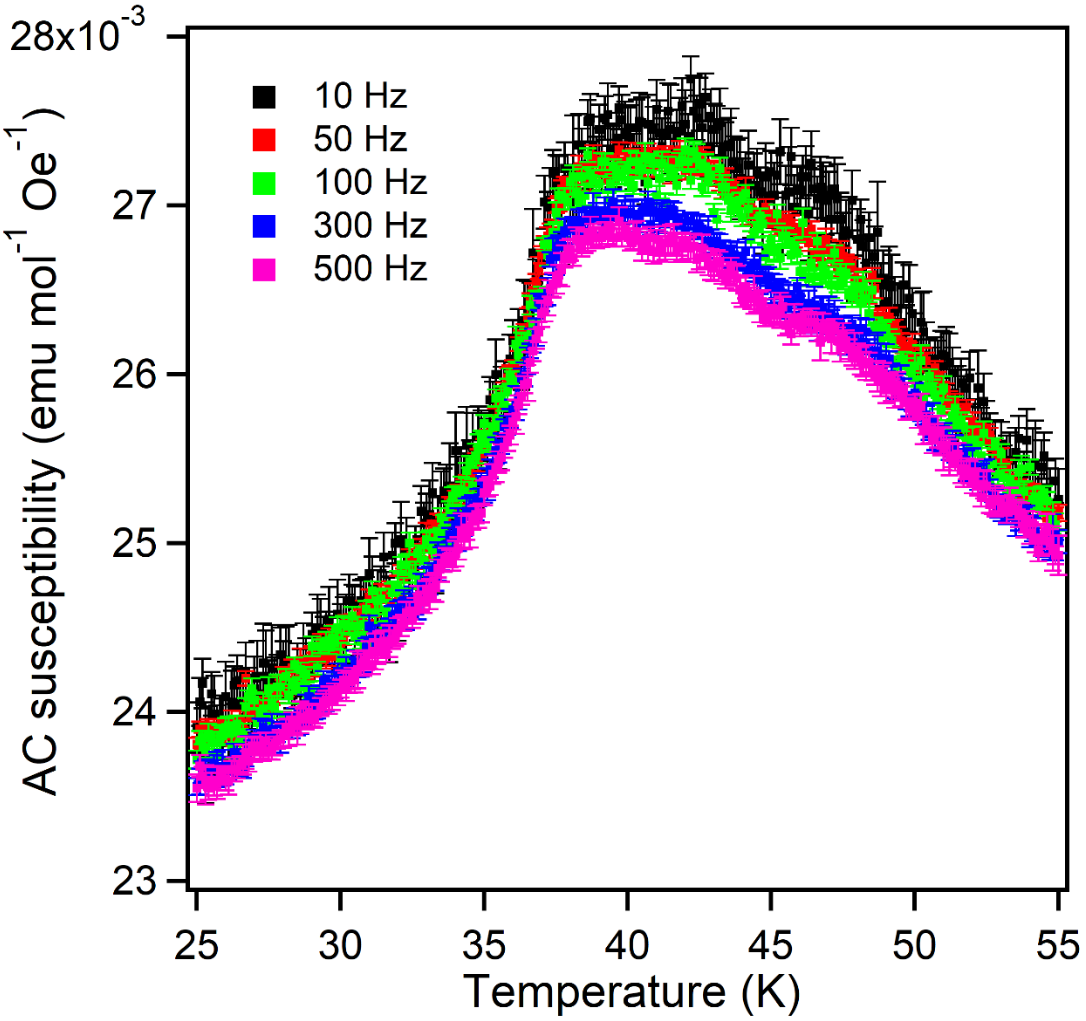

Figure S6. AC susceptibility collected from $FeCaTa_2O_7$ as a function of temperature at frequencies between 10 and 500 Hz.

Atom	Site	*x*	*y*	*z*	Occ.	B_{iso} / Å^2
Fe1	4*b*	0.8077(19)	0.7414(11)	0.26353(9)	1	1.80(4)
		M_x = 0	M_y = 0	M_z = 3.08(1)		
Ca1	2*a*	0.1106(35)	0.0073(19)	0	1	0.56(7)
Ca2	2*a*	0.5682(35)	0.5074(21)	0	1	0.56(7)
Ta1	4*b*	0.0586(30)	0.5091(11)	0.89530(24)	1	0.24(7)
Ta2	4*b*	0.5668(30)	0.9980(11)	0.11081(24)	1	0.24(7)
O1	2*a*	0.0939(33)	0.4371(12)	0	1	0.30(4)
O2	2*a*	0.4919(34)	0.0706(14)	0	1	0.30(4)
O3	4*b*	0.3218(31)	0.7381(12)	0.59201(19)	1	0.35(5)
O4	4*b*	0.2967(29)	0.2636(14)	0.42059(9)	1	0.35(5)
O5	4*b*	0.3308(30)	0.2548(14)	0.12481(10)	1	0.44(3)
O6	4*b*	0.3055(31)	0.7687(11)	0.90698(18)	1	0.44(3)
O7	4*b*	0.0274(32)	0.5298(14)	0.79425(39)	1	0.74(4)
O8	4*b*	0.5851(31)	0.9687(16)	0.20993(41)	1	0.74(4)

$FeCaTa_2O_7$ - Space group $P2_1n'm'$ (#31.127)

a = 5.51195(9) Å, *b* = 5.51556(9) Å, *c* = 18.60627(19) Å

volume = 565.658(15) Å^3

Formula weight = 569.81 g mol^{-1}, Z = 4

Radiation source: Neutron Time of Flight, Instrument: WISH

Temperature: 1.5 K

R_p = 5.82 % $_wR_p$ = 6.16 %

Table S7. Parameters from the structural and magnetic refinement of $FeCaTa_2O_7$ against NPD data collected at 1.5 K.

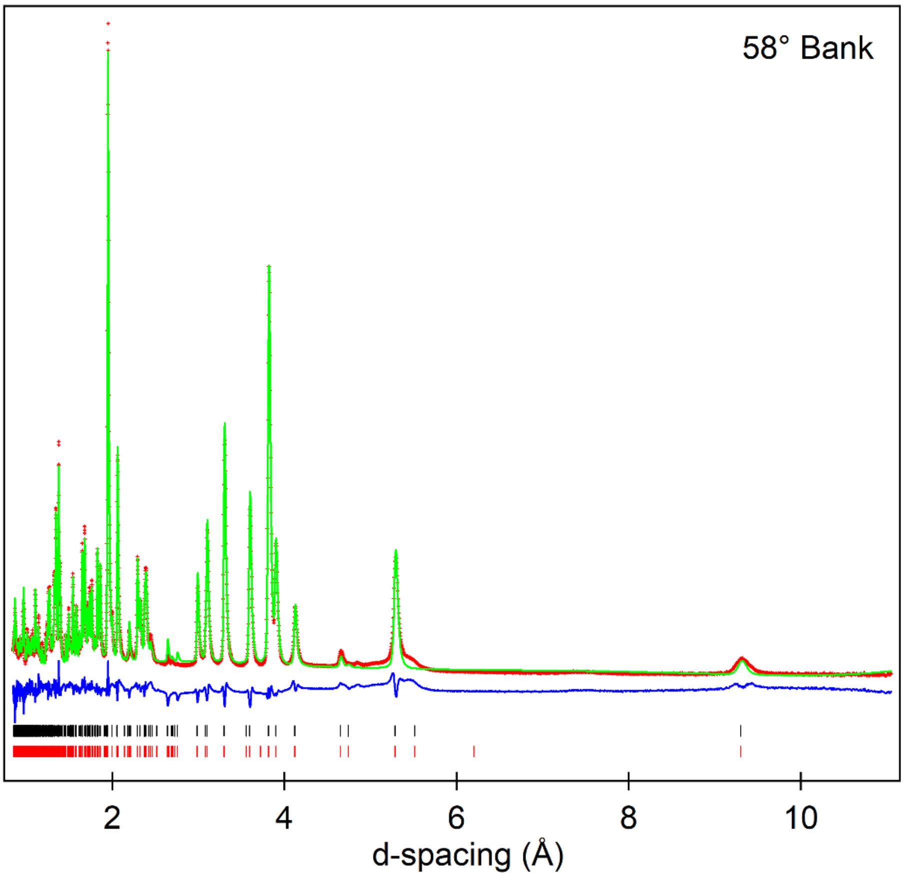
58° Bank
2
4
6
8
10
d-spacing (Å)

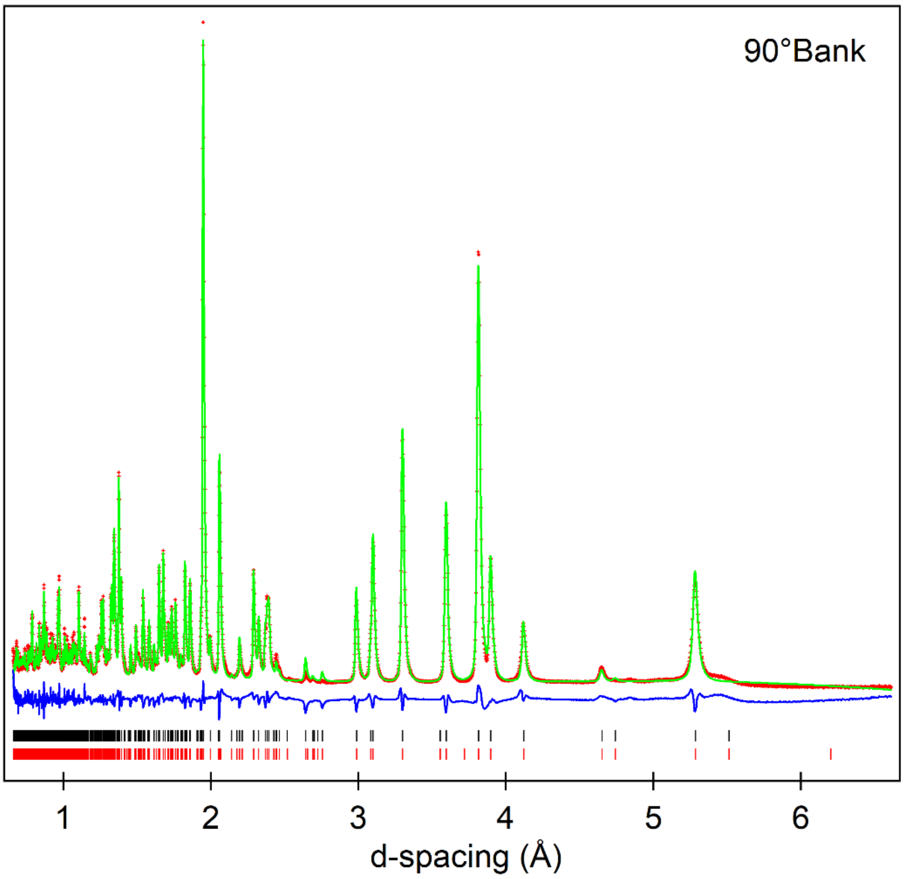
90°Bank
1
2
3
4
5
6
d-spacing (Å)

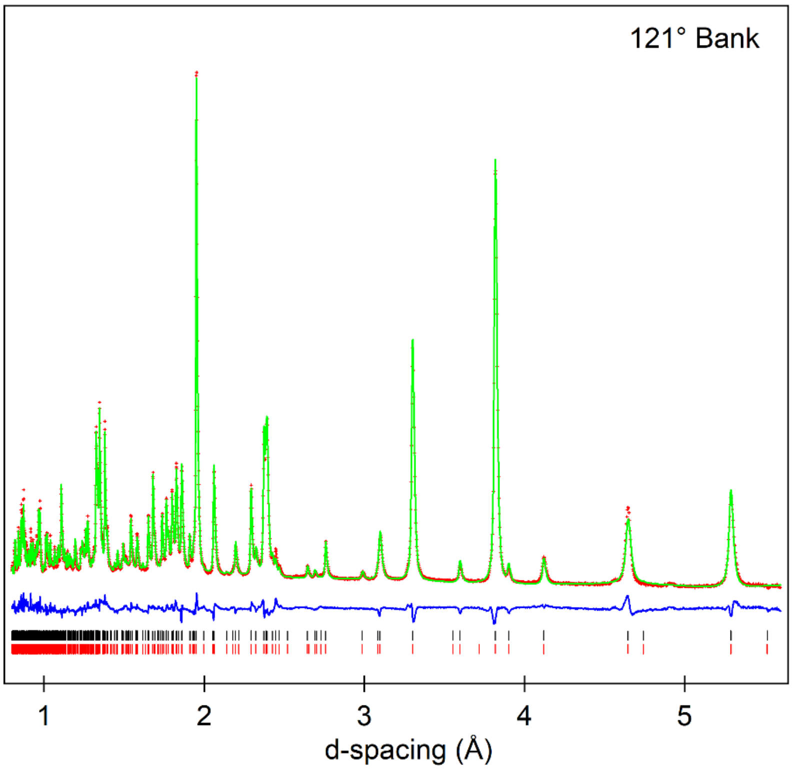

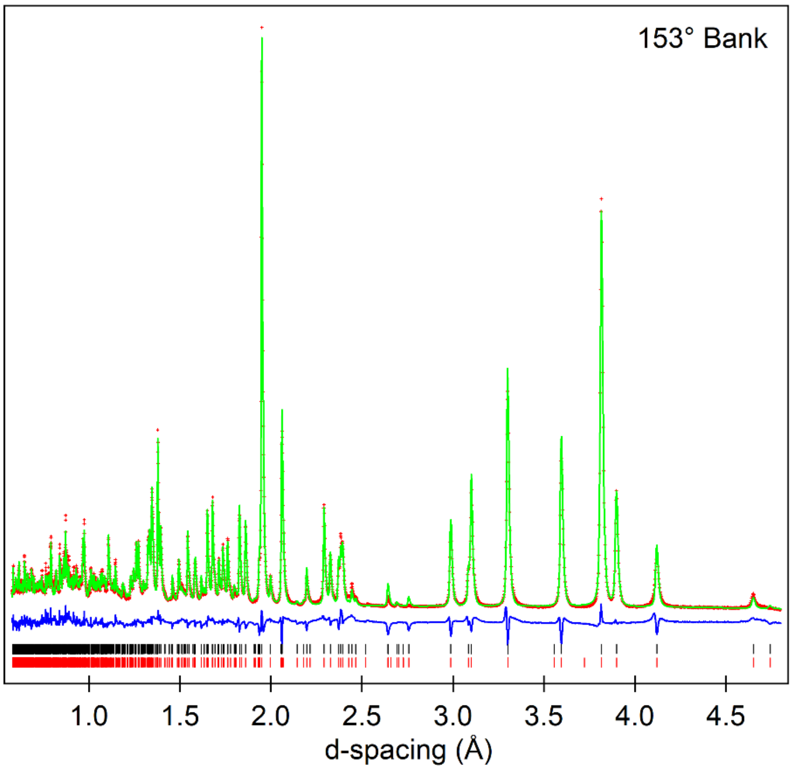

Figure S7 Observed calculated and difference plots from the structural and magnetic refinement of $FeCaTa_2O_7$ against NPD data collected at 1.5 K from the 4 different detector banks of the WISH instrument. Black ticks indicate nuclear peak positions, red ticks, magnetic peak positions.

6. **Magnetic Characterisation of $CoCaTa_2O_7$.**

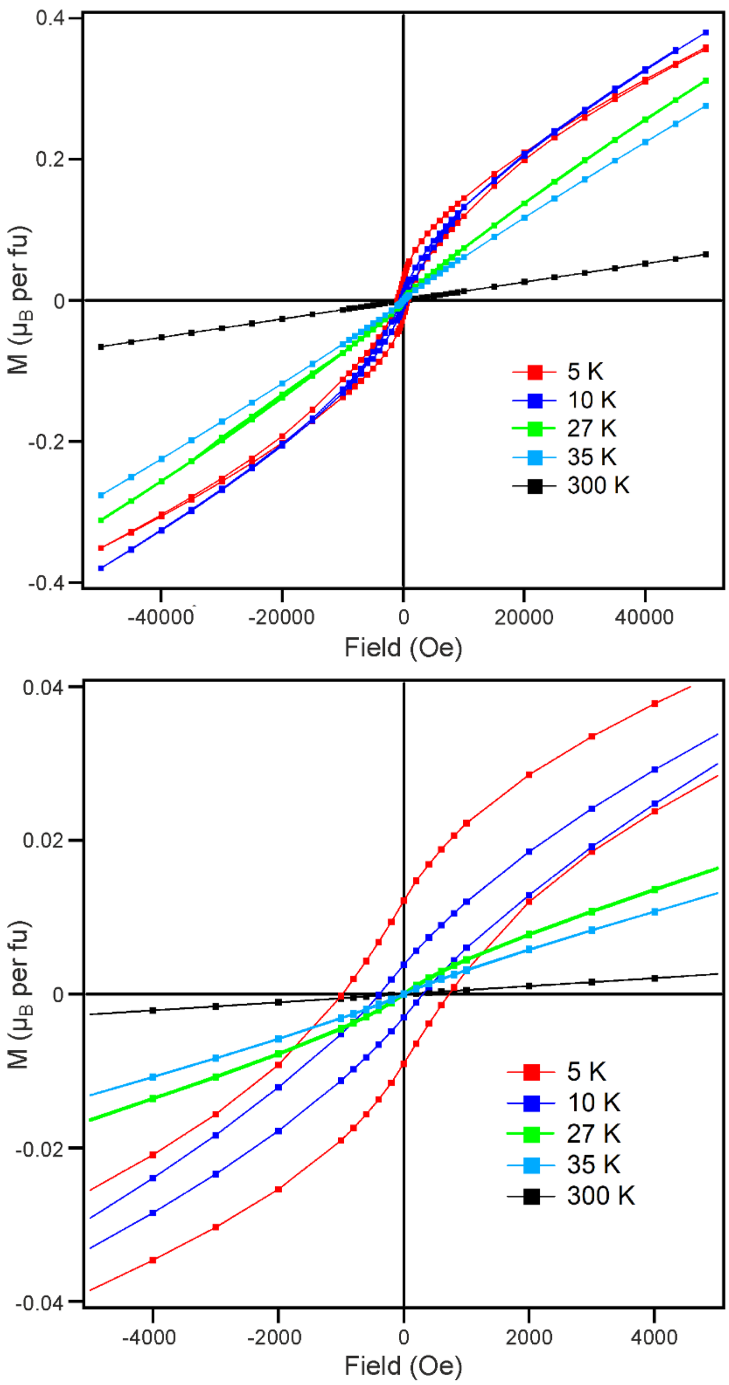

Figure S8. Magnetisation-field data collected from $CoCaTa_2O_7$. Lower panel shows expanded view around zero applied field.

Atom	Site	*x*	*y*	*z*	Occ.	B_{iso} / Å^2
Co1	4*b*	0.820(11)	0.7620(12)	0.2331(6)	1	5*
		M_x = 0	M_y = 0	M_z = 3.05(2)		
Ca1	2*a*	0.057(14)	0.9767(28)	0	1	0.75(6)
Ca2	2*a*	0.557(14)	0.5106(32)	0	1	0.75(6)
Ta1	4*b*	0.066(14)	0.4975(10)	0.8908(3)	1	0.35(4)
Ta2	4*b*	0.557(14)	0.9973(12)	0.1070(3)	1	0.35(4)
O1	2*a*	0.984(14)	0.5523(16)	0	1	0.91(5)
O2	2*a*	0.583(14)	0.9194(16)	0	1	0.91(5)
O3	4*b*	0.302(14)	0.7461(21)	0.5889(2)	1	0.64(4)
O4	4*b*	0.321(14)	0.2509(20)	0.3719(1)	1	0.64(4)
O5	4*b*	0.302(14)	0.2387(19)	0.0775(1)	1	0.59(4)
O6	4*b*	0.321(14)	0.7437(17)	0.9020(2)	1	0.59(4)
O7	4*b*	0.090(14)	0.4444(15)	0.7924(5)	1	0.98(4)
O8	4*b*	0.511(14)	0.0426(19)	0.2047(6)	1	0.98(4)

$CoCaTa_2O_7$ - Space group $P2_1n'm'$ (#31.127)

a = 5.51487(14) Å, *b* = 5.51863(14) Å, *c* = 18.58723(22) Å

volume = 565.693(21) Å^3

Formula weight = 572.90 g mol^{-1}, Z = 4

Radiation source: Neutron Time of Flight, Instrument: WISH

Temperature: 1.5 K

R_p = 4.04 % $_wR_p$ = 5.01 %

Table S8. Parameters from the structural and magnetic refinement of $CoCaTa_2O_7$ against NPD data collected at 1.5 K. Displacement factor of Co fixed at 5 Å^2, as this could not be refined satisfactorily due to weak scattering power of Co.

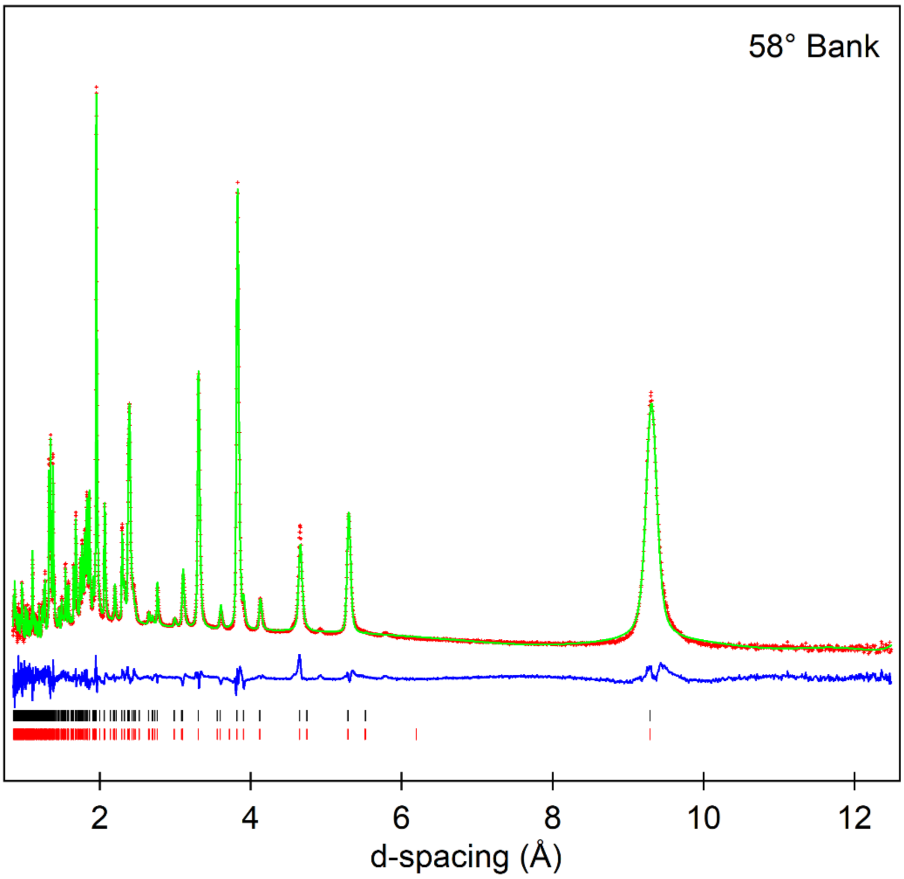

58° Bank
2
4
6
8
10
12
d-spacing (Å)

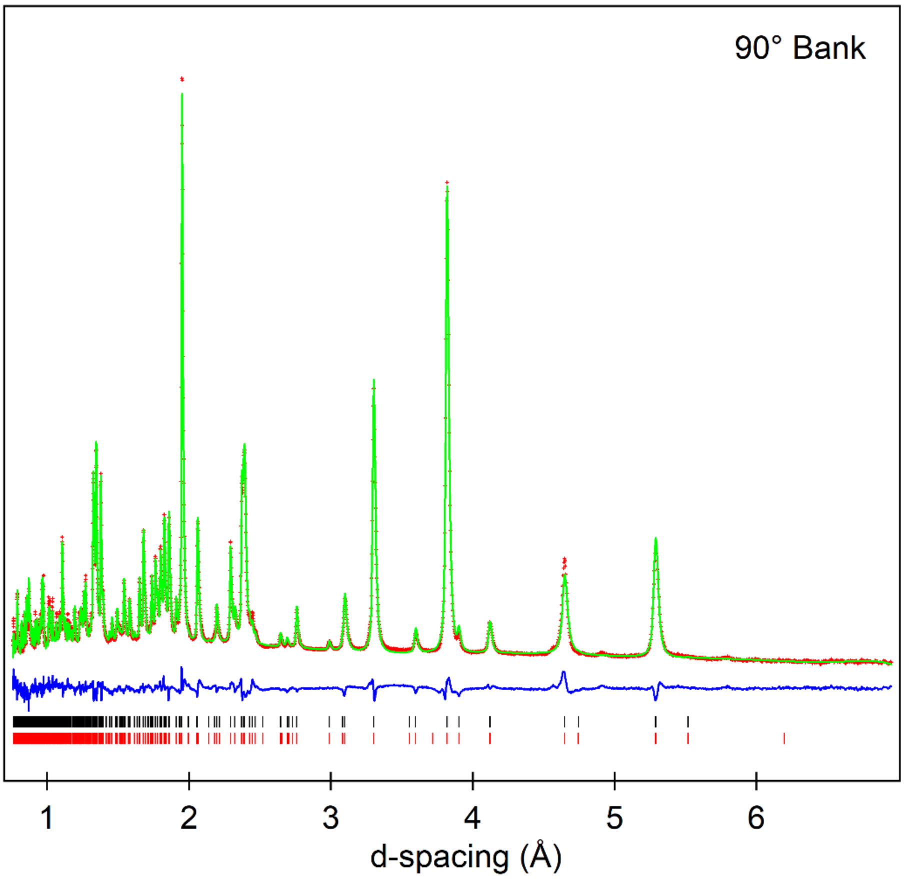

90° Bank
1
2
3
4
5
6
d-spacing (Å)

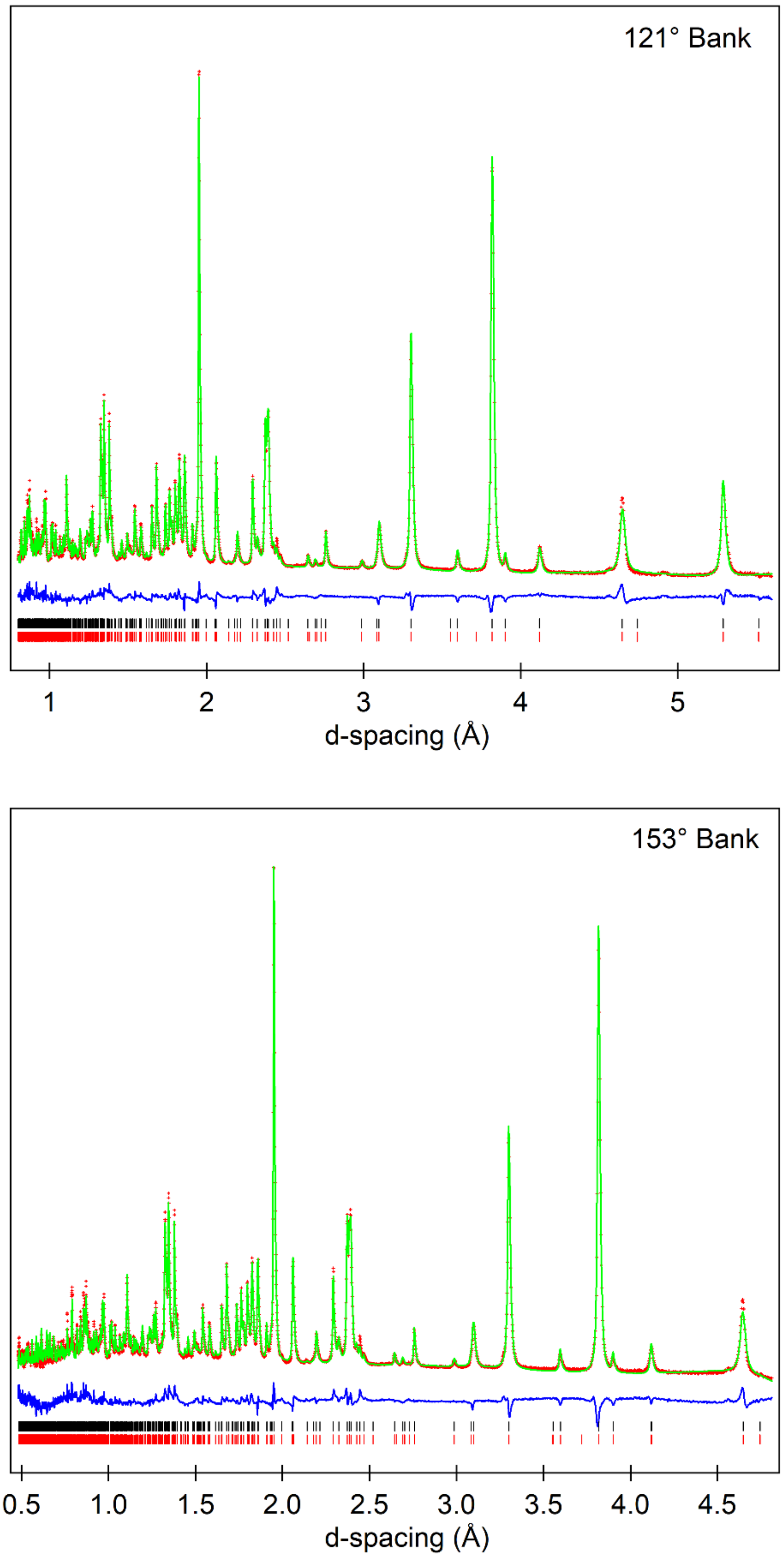

Figure S9. Observed calculated and difference plots from the structural and magnetic refinement of $CoCaTa_2O_7$ against NPD data collected at 1.5 K from the 4 different detector banks of the WISH instrument. Black ticks indicate nuclear peak positions, red ticks, magnetic peak positions.

7. Phase transition behaviour

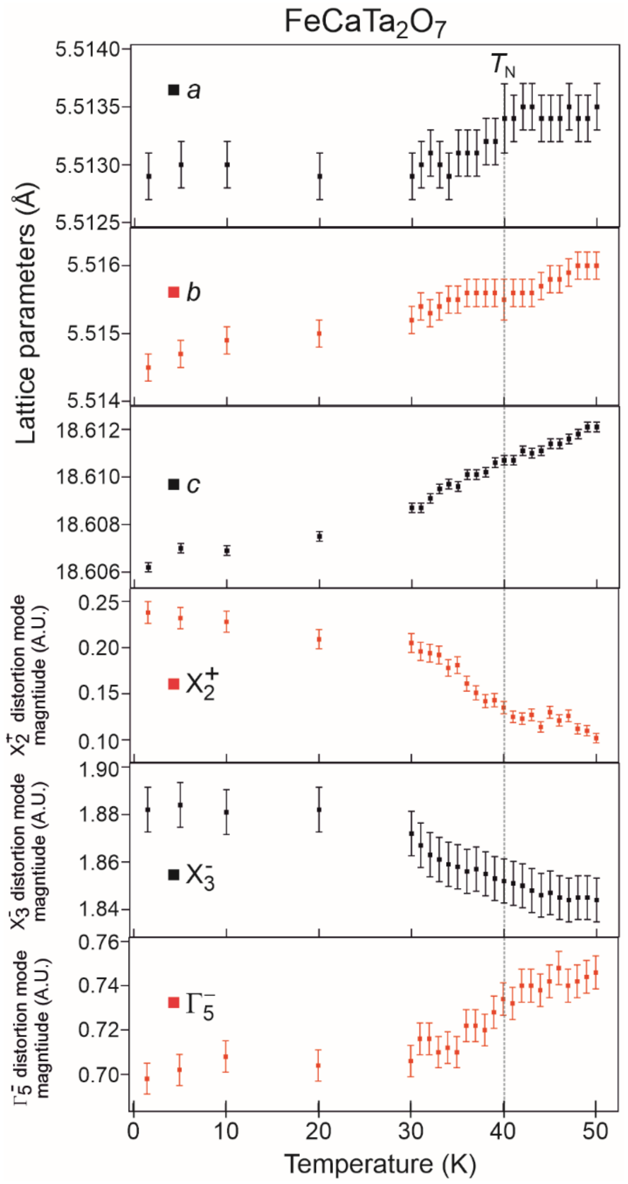

Figure S10. Lattice parameters, X_2^+ (0, *a*), X_3^- (*b*, *c*) and Γ_5^- distortion mode magnitudes of $FeCaTa_2O_7$ plotted as a function of temperature.

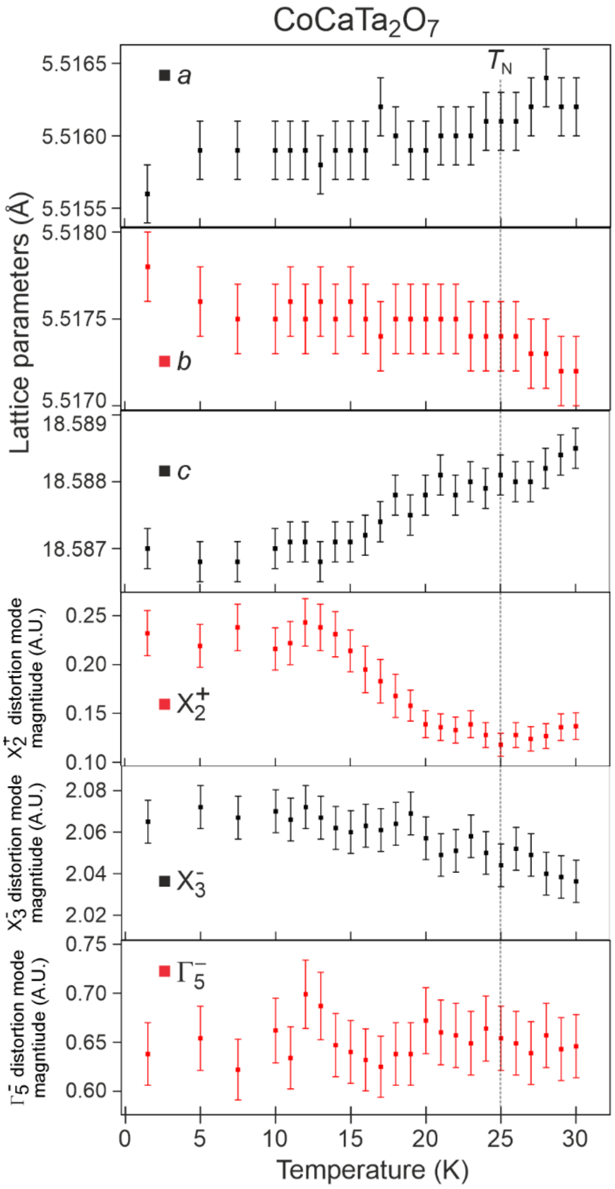

Figure S11. Lattice parameters, X_2^+ (0, *a*), X_3^- (*b*, *c*) and Γ_5^- distortion mode magnitudes of $CoCaTa_2O_7$ plotted as a function of temperature.
